# Mapping lymphatic filariasis in *Loa loa* endemic health districts naïve for ivermectin mass administration and situated in the forested zone of Cameroon

**DOI:** 10.1186/s12879-020-05009-3

**Published:** 2020-04-16

**Authors:** Andrew A. Beng, Mathias E. Esum, Kebede Deribe, Abdel J. Njouendou, Patrick W. C. Ndongmo, Raphael A. Abong, Jerome Fru, Fanny F. Fombad, Gordon T. Nchanji, Glory Amambo, Narcisse T. V. Gandjui, Benjamin Biholong, Georges Nko’Ayissi, Patrick Mbia, Julie Akame, Peter I. Enyong, Steven D. Reid, Jean J. Tougoue, Yaobi Zhang, Samuel Wanji

**Affiliations:** 1 Department of Microbiology and Parasitology, Parasites and Vector Biology Research Unit (PAVBRU), University of Buea, Buea, Cameroon; 2 Research Foundation in Tropical Diseases and the Environment (REFOTDE), Buea, Cameroon; 3 Centre for Global Health Research, Brighton and Sussex Medical School, Brighton, BN1 9PS UK; 4 School of Public Health, College of Health Sciences, Addis Ababa University, P.O. Box 9086, Addis Ababa, Ethiopia; 5 Ministry of Public Health, Yaoundé, Cameroon; 6 Helen Keller International, Yaoundé, Cameroon; 7 Helen Keller International, New York, NY USA; 8 RTI International, Washington, DC USA; 9 Helen Keller International, Regional Office for Africa, Dakar, Senegal

**Keywords:** *Wuchereria bancrofti*, FTS test, qPCR, *Loa loa*, Ivermectin, Forested zone

## Abstract

**Background:**

The control of lymphatic filariasis (LF) caused by *Wuchereria bancrofti* in the Central African Region has been hampered by the presence of *Loa loa* due to severe adverse events that arise in the treatment with ivermectin. The immunochromatographic test (ICT) cards used for mapping LF demonstrated cross-reactivity with *L. loa* and posed the problem of delineating the LF map. To verify LF endemicity in forest areas of Cameroon where mass drug administration (MDA) has not been ongoing, we used the recently developed strategy that combined serology, microscopy and molecular techniques.

**Methods:**

This study was carried out in 124 communities in 31 health districts (HDs) where *L. loa* is present. At least 125 persons per site were screened. Diurnal blood samples were investigated for circulating filarial antigen (CFA) by FTS and for *L. loa* microfilariae (mf) using TBF. FTS positive individuals were further subjected to night blood collection for detecting *W. bancrofti*. qPCR was used to detect DNA of the parasites.

**Results:**

Overall, 14,446 individuals took part in this study, 233 participants tested positive with FTS in 29 HDs, with positivity rates ranging from 0.0 to 8.2%. No *W. bancrofti* mf was found in the night blood of any individuals but *L. loa* mf were found in both day and night blood of participants who were FTS positive. Also, qPCR revealed that no *W. bancrofti* but *L.loa* DNA was found with dry bloodspot. Positive FTS results were strongly associated with high *L. loa* mf load. Similarly, a strong positive association was observed between FTS positivity and *L loa* prevalence.

**Conclusions:**

Using a combination of parasitological and molecular tools, we were unable to find evidence of *W. bancrofti* presence in the 31 HDs, but *L. loa* instead. Therefore, LF is not endemic and LF MDA is not required in these districts.

## Background

Lymphatic filariasis (LF) is a chronic, debilitating vector-borne disease caused by the filarial parasites *Wuchereria bancrofti, Brugia malayi* and *B. timori*. It is transmitted by *Culex*, *Anopheles* and *Mansonia* mosquitoes respectively [1]. In 1997, the World Health Organization (WHO) targeted LF for elimination by 2020 through a strategy of mass drug administration (MDA) [2, 3]. By the year 2000, WHO reported that, nearly 1.4 billion people in 73 countries worldwide were at risk of LF, with an estimated number of 120 million people infected, and about 40 million people disfigured and incapacitated by the disease [4]. Based on recent WHO reports [5], LF elimination as a public health problem was validated in several countries and 893 million people in 49 countries worldwide remain threatened by lymphatic filariasis and require preventive chemotherapy.

In order to carry out MDA, LF must be mapped to delineate areas where MDA is required and preventive chemotherapy (PC) given to the eligible population (in areas where prevalence of antigenemia is ≥1%), and with a minimum therapeutic coverage of 65% for 5–6 years [6]. The global strategy is a yearly single dose of two-drugs regiment, distributed to at-risk populations In Africa, WHO recommends an annual dose of ivermectin (150 μg/kg body weight) combined with albendazole (400 mg) due to the co-endemicity of LF and onchocerciasis in this continent [7].

LF was previously mapped in Cameroon using two strategies in two different zones. In the northern zone (two regions), which were not endemic for loiasis, *W. bancrofti* microfilaremia was confirmed microscopically using night blood smears [2]. In the southern part (8 regions) endemic for loiasis, LF was mapped based on the positivity of the immunochromatographic test (ICT) [8]. A total of 158 health districts in Cameroon were previously identified as endemic for LF. About 134 HDs were eligible for LF MDA following completion of epidemiological mapping and based on historical data [2, 8]. The other 24 health districts not eligible for MDA were later on carved out into 31 health districts by the health authorities and they were highly endemic for loiasis.

The implementation of MDA against LF in Cameroon started in 2008 in the North and Far North regions [9]. However, the implementation of MDA in the southern parts of Cameroon is facing serious drawbacks due to the co-endemicity of *Loa loa* and the risk of severe adverse events (SAE) in individuals with high microfilaria (Mf) loads, if treated with ivermectin [10, 11]. In the past, epidemiological mapping used ICT cards to assess the prevalence of LF but recent findings in Democratic Republic of the Congo (DRC) and Cameroon reported cross reactivity of this tool with high *L loa* Mf load in blood [12–14]. Another study demonstrated the loss of sensitivity of the ICT test in detecting *W. bancrofti* in low prevalence settings [15, 16]. Recently, a new strategy was developed to assess LF in co-endemic areas with other filariae [17].

To verify LF endemicity in forested areas in Cameroon where mass drug administration has not been started, this new strategy was applied using a combination of serological (FTS), parasitological (TBF) and molecular biological (qPCR) techniques.

## Methods

### Study sites

**Fig. 1:**
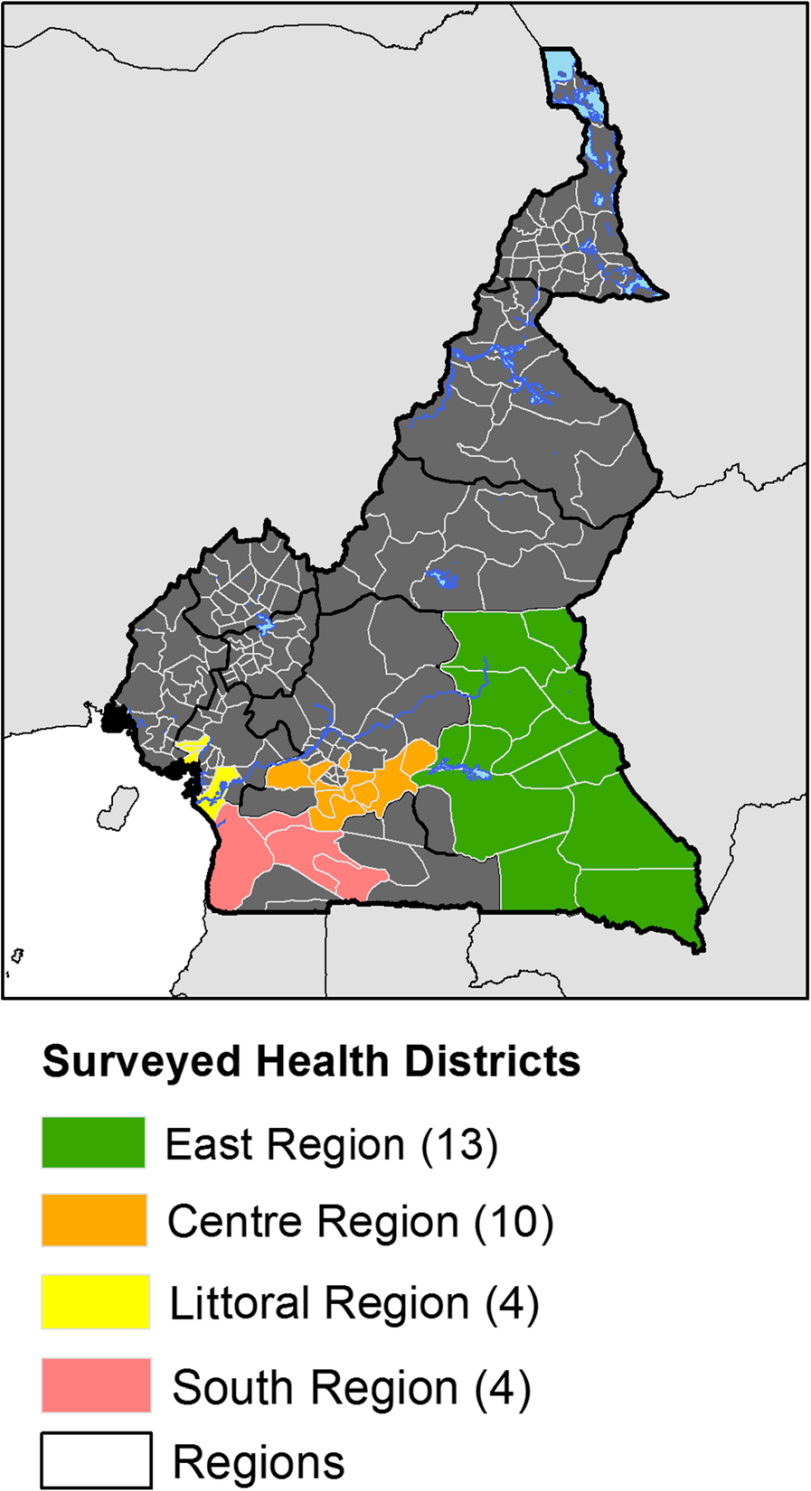
Map of the study area

The study was carried out in 31 health districts, located in 4 Regions in the forest zone of Cameroon. Thirteen health districts were surveyed in the East, 10 health districts were in the Center Region, four health districts each were in the South and Littoral Regions respectively. A total of 124 communities were involved, 4 from each of the 31 health districts. (Fig. 1; Supplementary material Figures S1 and S2). These communities are located in the forest area of Cameroon which are breeding sites of different filarial transmitting vectors and MDA has not been introduced in these communities. The survey was conducted from July 2016 to January 2017.

### Study design

This study was a cross-sectional community-based survey. Following mass sensitization on the importance of the study, a complete census of all households was carried out in each community and 50 households selected using simple random sampling. A minimum of 2 participants were randomly selected from each of the selected households.

**Fig. 2:**
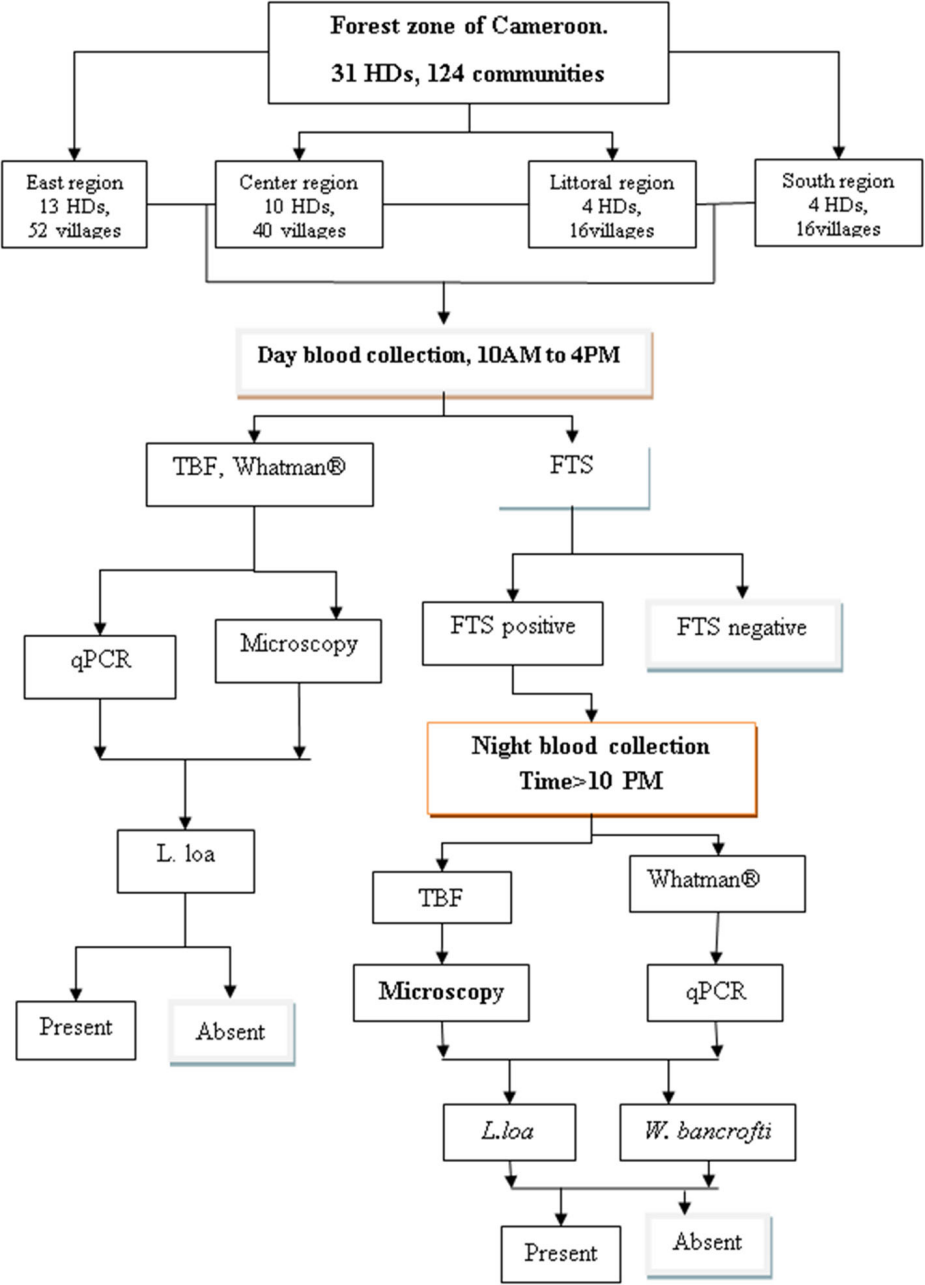
The algorithm of the study design

In each community, at least 125 participants both male and female of greater than 5 years, who had been resident in the community for at least 5 years, were recruited and screened during the day for the presence of *W. bancrofti* using the Alere Filarial Test strip (FTS, Alere, Scarborough, ME, USA). All eligible participants in the community who willingly consented or accented (if below 21 years) were recruited for the study. Sociodemographic factors were collected using a structured questionnaire. We used the algorithm shown in Fig. 2 to detect the presence of *W. bancrofti.* Blood samples for FTS testing, thick blood film (TBF) and dry blood spot (DBS) for real time polymerase chain reaction (qPCR) were also obtained. Night blood samples (10:00 pm to 12.30 am) was collected from FTS positive individuals for microscopy to detect *W. bancrofti* microfilariae, and for qPCR.

### Circulating filarial antigen test with FTS

Antigen testing was performed with FTS according to the manufacturer’s instructions. In brief, 75 μL of finger prick blood collected from eligible participants using non-heparinized microcapillary tubes (soda lime glass, Modulohm A/S Herlev, Denmark) was tested using FTS and the test was allowed to run for 10 min before being read. The result was recorded on the data record sheet. Individuals who tested either negative or positive on the FTS were informed of the result and those who were positive asked to return at night between 10:00 PM – 12:30 AM to have additional blood taken for microscopic evaluation of *W. bancrofti* mf. It was necessary to have the blood taken at night due to the nocturnal periodicity of *W. bancrofti* mf in the blood. Quality controls for the FTS strip were conducted daily before the exercise, using positive and negative controls from the manufacturer, to ascertain standards and kits performance throughout the study.

### TBF for microscopy

Standardized 50 μL of blood was collected with a non-heparinized microcapillary tube to identify mf of *W. bancrofti* (night blood of FTS positive individuals, between 10:00 pm to 12.30 am) or mf of *L. loa* (day blood, 8 am to 4 pm)**.** In brief, the collected 50 μL of blood was placed on the centre of a clean slide, and spread repeatedly in a circular area of about 1.5 cm using the microcapillary tube. They were air dried and packaged for staining at the base. All blood smears were stained with 10% Giemsa within 24 h. The stained smears were examined using a light microscope at 10X objective lens (or using 40X objectives lens), for blood dwelling mf. Any mf present were identified based on the the size and presence or absence of a sheath, quantified and recorded.

### Whatman dry blood spot (DBS)

For each participant, 6 spots of 50 μL of blood were loaded onto Whatman filter paper (GE Healthcare UK Ltd., Little Chalfont, United Kingdom.) to prepare dry blood spots (DBS) for analysis by qPCR. After drying, DBS were kept separately in individual plastic bags at ambient temperatures and was later stored at − 80 °C until processed. Given the diurnal periodicity of *L. loa* mf, sampling was performed between 10:00 AM and 4:00 PM. DBS were also collected for all individuals who were FTS positive during night blood sample collection as described above.

### Detection of filarial DNA in DBS by qPCR

DNA was extracted from the DBS, using the QIAGEN DNeasy kit (QIAGEN, Valencia, CA) following the manufacturer’s instructions. Briefly, one DBS of a participant was cut out and placed in a 2 ml microtube, covered with 270 μL of ATL buffer, incubated at 85 °C for 10 minutes, then at 56 °C for 1 hour after addition of 30 μL of proteinase K. Addition of 300 μL of Al buffer to the digested suspension brought up the lysate volume to a total of 600 μL. From this step, the lysates were heat-treated (100 °C), prior to the DNA purification, to denature the genomic DNA to make the DNA target sequence more accessible to the primers. A volume of ethanol equivalent to half the volume of lysate sample was added before loading the mixture onto a DNeasy spin column. Depending on the volume loaded, additional centrifugations were performed to pass all the solution through the column. After washing, (twice with AW1 buffer, once with AW2 buffer), purified DNA was eluted in 200 μL of AE buffer. The qPCR assays for *W. bancrofti* were performed using 2 μL of DNA and the *W. bancrofti-*specific long DNA repeat (LDR) primers as described by Rao et al. in 2006 [18]. For the detection of *W. bancrofti* the “long DNA repeat” (LDR) was used as a target. The nucleotide sequences for the forward and reverse primers were LDR 1, 5′ ATTTTGATCATCTGGGAAGGTTAATA 3′ and LDR 2, CGACTGTCTAATCCATTCAGAGTGA3 and the sequence for the probe was /56FAM/ATCTGCCCA/ZEN/TAGAAATAACTACGGTGGAT CTCTG/3IABkFQ. All assays were performed in duplicate using kappa probes master mix kit (Kappa Biosystems, Wilmington, MA) with 20 pmol of each primer (LDR1 and LDR2) and 6 pmol of LDR probe per well in the final volume of 20 μL and the fast PCR programmed automatically (95 °C temperature 40 cycles for 20 s, 60 °C for 1 s and 72 °C for 20 s) in a Step-One-Plus PCR system (Applied Biosystems, Foster City). The primers for *L. loa* PCR were LLMF72, 5’CGGAAGACTCAACGTCAGAAATCA3’ and 5’AGGAACGCTGATGGTGATGT3 and for the probe was /56FAM/CCAACAGCC/Zen/TGCTTT/31ABkFQ. qPCR were performed to identify DNA from *L.loa* mf as described by Fink et al. [19]. These entire tests were performed with 1 μL of extracted DNA (representing 0.1427 μL of whole blood).

### Data management

Data generated either in the field or laboratory were collected using smart phones and uploaded to a center server coded with a password at the end of each day, to prevent accidents or the phones getting bad. The data were compiled and managed using Epi Info version 7.2 (Center for Disease Control and Prevention, Atlanta, GA) and Microsoft Excel 2013. It was checked for missing values and redundancy. All missing values were registered as missing.

After cleaning, the data was re- uploaded to the main server and hard copies in DVDs were also made. All hardware and software carrying the research data can only be accessed by the research team. Security password and server cupboard had been set up to protect the data at the University of Buea/REFOTDE, Buea, Cameroon.

### Data analysis

Data were analyzed using the IBM SPSS Statistics (version 20.0, IBM, Armonk, NY, US). Some descriptive graphs were drawn with MS Excel 2010 and Thematic analysis were performed using the ArcGIS software (version 10.2, ESRI Inc. Redlands, CA, US), to draw the LF map in the 31 health districts and other maps in the analysis (Using GPS coordinates from each study community). The differences across age and gender were tested using fishers statistics and the 95% confidence interval (CI) computed. Chi-square, Mann-Whitney and Kruskal-Wallis tests were also used to compare LF and *L. Loa* prevalence level, the Geometric mean index of infection between regions and HDs, sex and age groups respectively. To assess the relationship between FTS positivity and *L. loa* Mf loads, the geometric mean intensity (GMI) of Mf counts was calculated as follows: GMI = (∑log(x + 1)/n),Where X = the number of Mf per ml of blood in Mf positive individuals, n = the number of mf positive individuals. Spearman rho correlation analysis was carried out between FTS positivity rate and TBF data (*L.loa* Mf load) at the regional level to find out if there was any relationship. Odds ratios were calculated to quantify the risk of an individual harboring *L. loa* Mf and testing positive to FTS compared to the risk for an amicrofilaraemic individual. The value was used to quantify the risk or the relation established by the correlation statistics. Logistic regression analysis was used to determine the predictors of FTS positivity. Gender, sex and quantity of *L. loa* Mf load were cross tabulated with FTS positivity.

The base map of the global administrative areas was downloaded from the Natural Earth (https://www.naturalearthdata.com/) [20]. All maps were produced using ArcGIS Desktop v10.5 (Environmental Systems Research Institute Inc., Redlands CA, USA).

### Ethical consideration

Ethical approval for this work was obtained from the Cameroon National ethics committee (CNEC) number 2015/09/640/CE/CNERSH/SP of 16th September 2015. The Administrative approval number D112-244NS/MINSANTE/SG/DLMEP/SDL/PMDTN/PNLO of 11th august 2016, was granted by the Ministry of Public Health of Cameroon. The investigators ensured that this study was carried out according to the current revision of the Helsinki Declaration and Good Clinical Practices regulations and guidelines from the International Conference of Harmonization (ICH-GCP). Moreover, the investigators ensured that all protocol activities were guided by the ethical principles of the Belmont Report, 45 CFR 46, and all its subsections (A, B, C and D). All ethical issues like; handling of withdrawals, benefits and risks, *community participant risk with finger prick, biohazard risk to field technicians, risk associated participant confidentiality and test results,* reporting adverse events and unanticipated problems, confidentiality, etc., as stipulated in the study protocol were strictly observed. The purpose of the study was explained to the community leaders and the study participants in their local languages at the time of recruitment. Individual informed written consent was obtained from each participant. If a participant was less than 21 years old (the age of majority in Cameroon), written assent and permission were obtained from the study participant and a legal guardian respectively. The data was analyzed and reported to exclude any directly identifiable information in order to maintain the anonymity of the participants.

### Clinical examination

Trained medical personnel recruited for this study, examined all the participants for lymphedema. All males were examined for signs of the limb lymphedema and hydrocele and female for limb lymphedema. The lymphedema cases also underwent night blood collection for circulatory filarial antigen test and thick blood film microscopy.

## Results

### Study population characteristics

In all, 14,446 participants took part in this survey and underwent the FTS test. Of these, 49.99% (7221**/**14446) were males with a mean age of 31.8 ± 19.8 years and 50.01% (7225**/**14,446) were females, with a mean age of 32.1 ± 20.1 years. 4151 (28.7%) persons tested were less than or equal to 15 years and 10,295 (71.3%) persons tested were > 15 years (Supplementary material Table S1, Supplementary material Fig. S3).

### Prevalence of positive FTS

**Fig. 3:**
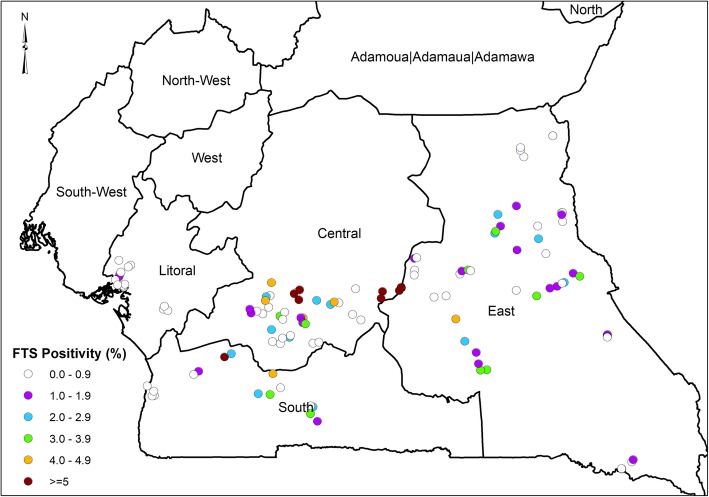
FTS Positivity across the different communities

Out of a total of 14,446 individuals screened, 233 tested positive with the FTS (Table 1). The overall prevalence of positive FTS was 1.6% (233/14446), ranging from 0.0% in Abo and Bonassama HDs (Littoral Region) to 8.2% in Ayos HD (Center Region) (Fig. 3 and Supplementary material Table S1). Among the 31 HDs surveyed, 18 HDs had a prevalence of FTS positive rates equaled to or greater than 1% (Supplementary material Table S3). The males had a significantly higher FTS positivity rate of 2.0% (141/7221) compared to the females 1.3% (92/7225) (Fischer’s statistics, *p* < 0.05). The FTS positivity between the age groups was also statistically significant, higher in adults (15 years and above) with 2.1% (218/10295) compared to 0.4% (15/4151) in children (< 15 years) (Supplementary material Table S2).

**Table 1:** Prevalence and intensity (GMI mf/ml) of *Loa loa* microfilarial for FTS positive individuals during day and night

REGION	Screened (N)	FTS Prev. (%)	DAY				NIGHT			
			FTS + ve Participants (N)	***L.loa*** +ve (N)	Loiasis Prev (%)	GMI (mf/ml)	FTS + ve Participants (N)	***L. loa*** +ve (N)	Loiasis Prev (%)	GMI (mf/ml)
**EAST**	**6444**	**1.4**	87	61	(70.1%)	621.3	*68*	*44*	*(64.70%)*	*61*
**CENTER**	**4475**	**2.4**	109	65	(59.60%)	210	*97*	*50*	*(51.50%)*	*18.9*
**LITTORAL**	**1789**	**0.3**	5	1	(20.0%)	5.9	*2*	*0*	*(0.0%)*	*0*
**SOUTH**	**1738**	**1.8**	32	20	(62.5%)	344.3	*21*	*15*	*(71.4%)*	*44.6*
**TOTAL**	**14,446**	**1.6**	**233**	**147**	**63.1%**	**311**	***188***	***109***	***58%***	***30.9***

* + ve indicates positive, *GMI* geometric mean intensity, *Prev* prevalence, *N* number

In the Center Region, out of the 4475 participants that took part in the study, 2.4% (109/4475) were FTS positive (Fig. 3, Supplementary material Table S7). The prevalence in this region ranged from 0.5% (2/422) in Ngog Mapubi HD to 8.2% (37/452) in Ayos HD. The prevalence was higher in males 2.9% (62/2158), than in females 2.0% (47/2317), but the difference was not significant (*p* < 0.066). There was a significantly more positive FTS strips in adults 3.0% (101/3312) compared to children 0.7% (8/1163) (*p* < 0.001). (Supplementary material Table S3).

In the East region, a total of 6444 participants took part in the study with an overall prevalence of 1.4% (87/6444). The prevalence ranged from 0.2% (1/534) in Garoua Boulaye to 2.5% (12/481) in Lomie (Supplementary material Table S3). The 1.8% (12/481) prevalence among adults was significantly higher when compared to 0.3% (6/2005) in Children (p < 0.001). The distribution in females was lower than that of males, 1.1% (34/3147) and 1.6% (53/3297) respectively (*p* < 0.067) but not statistically significant.

A total of 1789 participated in the Littoral, with a general prevalence of 0.30% (5/1789), the FTS positivity ranged from 0.00% in Abo and Bonassama to 0.8% (3/397) in Dibombari. Here adults (> 15 years) were more FTS positive than children (< 15 years), with 0.40% (5/1261) and 0.00% (0/528) respectively but the difference was not significant (*p* < 0.334). Though not significant, males were more positive 0.40% (3/853) than females 0.20% (2/936) (*p* < 0.674) (Supplementary material Table S3).

In the South Region, a total of 1738 took part in the study recording a prevalence of 1.80% (32/1738). FTS positivity ranged from 0.60% (3/470) in Kribi to 2.7% (7/264) in Lolodorf. Males were more positive to FTS than females with 2.50% (23/913) and 1.10% (9/825) respectively (*p* < 0.031). Children were significantly less positive 0.20% (1/455) compared to adults 2.40% (31/1283) (*p* < 0.001) (Supplementary material Table S3).

### Prevalence of lymphedema

A total of one hundred and sixty three (163) lymphedema cases were diagnosed in the entire study population, giving a prevalence rate of 1.14% (Supplementary material Table S1 and Figure S4). None of them tested positive with FTS for *W. bancrofti* antigen. Mbang health district in the East region had the highest number of lymphedema cases 36/503 (7.2%) followed by Ayos in the Center region 20/452 (4.4%). Both males 1,1% (82/7221) and females 1.1% (81/7225) were affected almost equally with lymphedema.

### *L. loa* mf load in day thick blood films (TBF)

**Fig. 4:**
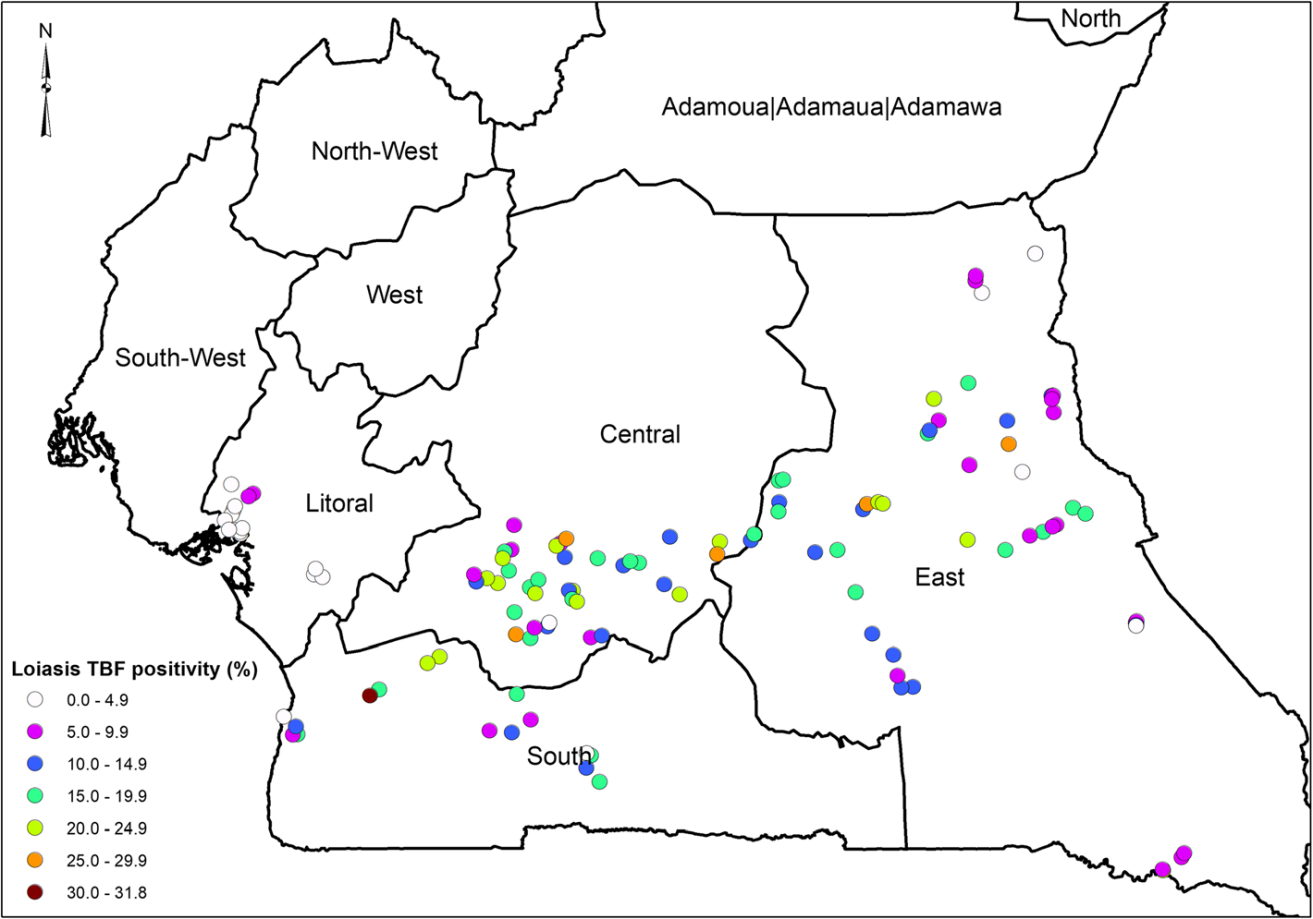
Distribution of positivity of *Loa loa* Mf load from TBF

*L loa* mf prevalence for the 31 health districts is shown on Supplementary material Tables 1 & 8. The overall prevalence of *L loa* mf from diurnal blood was 12.5% (1805/14442). The prevalence ranged from 2.7% (50/1789) in the Littoral to 16.6% (745/4475) in the Center region (Supplementary material Table S8). A high mf prevalence was also recorded in the East region 12.3% (793/6442). The prevalence among the males of 14.8% (1071/7221) was significantly higher than that in females of 10.2% (734/7225) (*p* < 0.001) (Supplementary material Table S2). Between the two age groups, adults with 15.8% (1624/10295) were significantly more infected than children with 4.4% (181/4151) (p < 0.001). The thematic map showing *L loa* mf distribution across the 31 health districts is shown on Fig. 4.

### Microfilaremia in FTS-positive individuals

We had 233 participants that were FTS positive. Microscopic results were available for all 233 for day time TBF and just 188 for night time TBF (Table 1). No *W. bancrofti* mf was found on any of these slides (day or night TBF). But, *L. loa* mf were present in 147/233 (63.1%) day blood TBF and in 109/188 (58%) night blood TBF (Supplementary material Table S9). The total mean mf load of *L. loa* (mf/ml) was 11,343 during the day and 2402 at night. The average *L. loa* mf GMI was 311 mf/ml during the day and 30.9 mf/ml during the night.

### Correlation between positivity of FTS and prevalence of *L*. *loa*

There was a positive correlation between the proportion of positive FTS and the prevalence of *L*. *loa* mf (Supplementary material Figure S5 and S7). In FTS positive individuals across the four regions, FTS positivity was significantly related to *L loa* mf load (*p* < 0.001) (Supplementary material Table S6), as the amount of *L loa* mf load increased, the probability of being FTS positive also increased, indicating that harboring *L loa* in the blood was a good predictor for having a positive FTS results.

The odd ratios of a person in any of the four regions with high *L loa* mf load of 8001–20,000 mf/ml being detected CFA positive by FTS was 53.2 times (OR = 53.2; 95% CI: 35.2–80.4; *p* < 0.001), compared to an individual in these regions with lower *L loa* mf load of 1–8000 mf/ml whose odds ratio to be detected CFA positive by FTS is just 6.1 (OR = 6.1; 95% CI: 4.4–8.6; p < 0.001). The OR becomes extremely high when mf load was > 30,000 mf/ml (OR = 151.9; 95% CI: 85.4–270.2; p < 0.001) (Supplementary material Table S4).

The logistic model also showed that gender is not associated with FTS positivity and *L. loa* prevalence (*p* < 0.076) but there was a contrast across age groups, adults had a significant risk of being FTS positive with high *L. loa* loads within these four regions with an OR of 3.395 times more than children (OR = 3.395; 95% CI: 1.942–5.935; *p* < 0.0001) (Supplementary material Table S10).

**Fig. 5:**
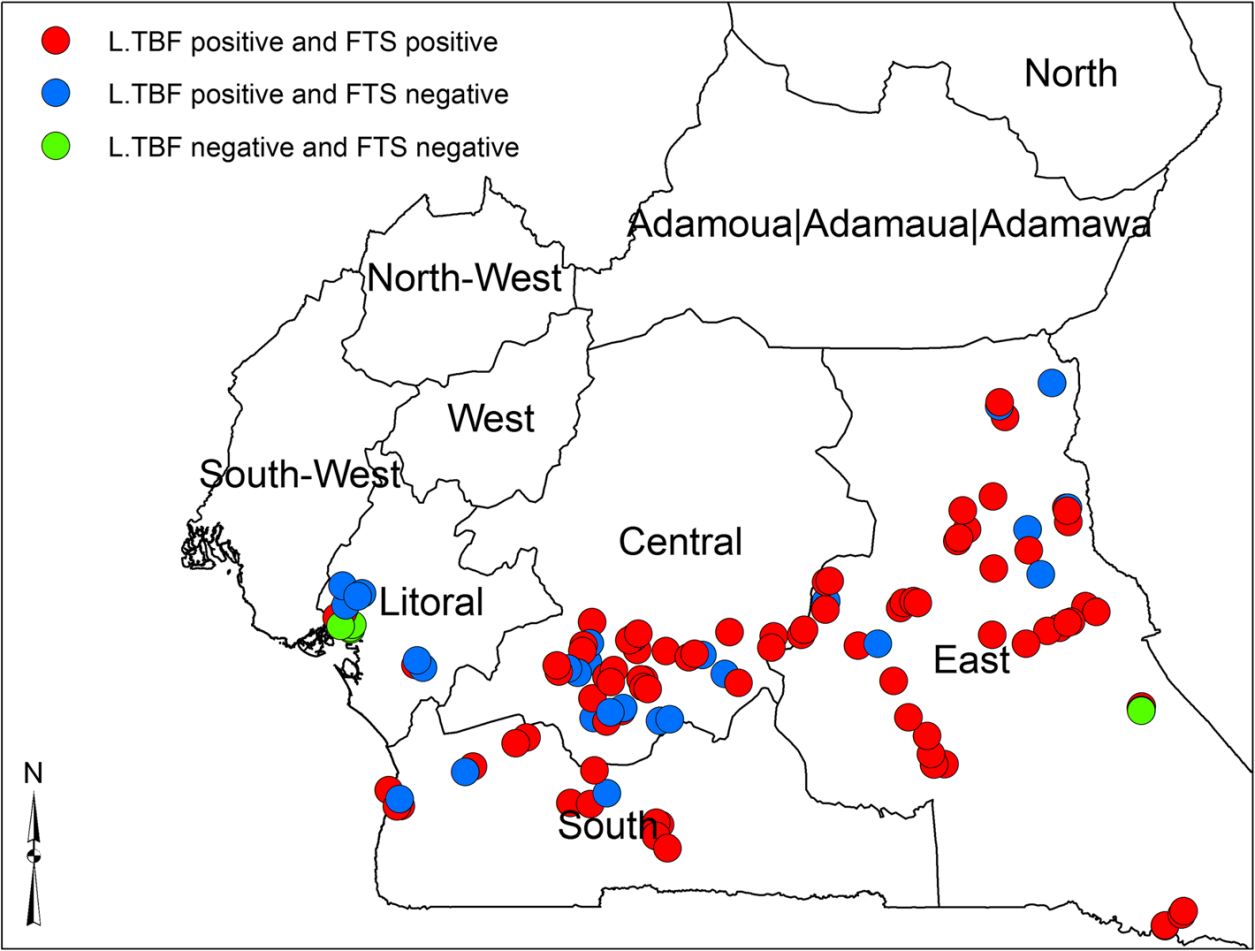
Concordance between the positivity Of FTS and positivity of TBF *Loa loa* in the surveyed Communities

In Supplementary material Figure S6 a plot of positive FTS and *L loa*, showed that every increase in *L loa* Mf prevalence is followed by an increased in FTS positivity. The concordance between FTS positivity and that of Thick blood film (TBF) prevalence of *L. loa* in the surveyed Communities is in shown in Fig. 5.

### Molecular analysis by qPCR

The detection of *W. bancrofti* DNA by qPCR was done for 182 of the 229 night blood DBS collected from the FTS positive individuals. All tested negative for LDR DNA sequence, specific to *W. bancrofti*. However, 104 of the 182 samples (57.1%) were positive for *L. loa* specific LLMF72 DNA sequence (Table 2 and Supplementary material Table S11).

**Table 2:** Comparing Molecular (qPCR) with Thick blood film (TBF) microscopy of FTS positive individuals

Region	FTS + ve participants (*N)	qPCR		TBF (Microscopy)	
		***W. bancrofti*** N(%)	***Loa loa*** + ve N(%)	***W. bancrofti*** N(%)	***Loa loa*** + ve N(%)
**EAST**	64	0	44 (68)	*0*	*43 (67.2)*
**CENTER**	92	0	48 (52.2)	*0*	*46 (50)*
**SOUTH**	23	0	12 (52.2)	*0*	*14 (60.9)*
**LITTORAL**	3	0	0 (0)	*0*	*0*
TOTAL	**182**	0	**104 (57.1)**	*0*	***103 (56.6)***
**Mc Nemar test:** p- value = 0.999					

*N number

There was no significant difference between the qPCR results and those obtained from microscopy (Wilcoxon *p* value *p* < 0.999). The qPCR and microscopy results were plotted to depict how these tests performed in each HD with Supplementary material Figure S8.

## Discussion

The FTS is the current generation filarial antigen test that was developed to improve on the ICT Card test for mapping of LF. Laboratory and field evaluation of the FTS carried out in areas with high residual LF endemicity after multiple MDA rounds, showed that FTS had significant technical and practical advantages over the ICT [21].

The prevalence of FTS positivity in the study area was 1.6%. This value is lower compared to 2.9 and 3.3%, obtained in other surveys in Cameroon using the ICT in the mapping of *W. bancrofti* [11, 13]. However, FTS positivity was much higher than the prevalence obtained by other previous works 1.1 and 0.4% [14, 22, 23]**.** There is concordance between FTS positivity and *L. loa* density and prevalence, which may indicates that FTS positivity is a result of *L. loa* endemicity in the study area. This may also reflect the geographical variations in the distribution of *L. loa.*

The FTS positivity in males 2.0% (141/7221) was higher than that for females 1.3% (92/7225) (*p* < 0.05), and this trend was previously observed in similar studies in Sri Lanka and Indonesia [21]. The results also showed that age was significantly associated with FTS positivity as adults turned to be more positive than children, but there was no significant association between gender and FTS positivity (*p* < 0.075)**.** The difference in FTS positivity within age groups, 2.1% (218/10295) above 15 years (adults) compared to 0.4% (15/4151) less than 15 years, showed that circulating filarial antigens (CFA) was increasing with age.

FTS positivity status at the district level showed that all but 2 of the 31 HDs had FTS non-reactive results, and 18 HDs had FTS positivity proportions ≥1%. Similar to other results observed with the ICT [13]. It was also observed that the level of mf/ml in blood for *L loa* also increased with an increase in age which further indicates an association between FTS positivity and *L loa* mf load. The results revealed that the prevalence of positive FTS falls within other reported ranges using ICT. The main predictor of this positivity, included high *L.loa* mf load in blood and to a lesser extend the age group, which was higher in adults > 15 years. Gender was not associated with FTS positivity but there was a strong association with high *L. loa* mf load > 8000 mf/ml of blood with high odds ratio of being FTS positives [11, 13]. Analysis with microscopy using night blood TBF and qPCR, indicated zero presence of *W. bancrofti* but a high *L loa* densit*y.* The absence of *W. bancrofti* in night blood smears and qPCR, confirm previous findings [15, 17], that high *L loa* mf was probably the cause of FTS positivity, as earlier observed with ICT due to cross-reaction, since FTS was developed with the same reagents as ICT [16].

Discrepancies were noticed between FTS results with those obtained from microscopy and qPCR respectively. However, previous works [15] reported high *L loa* mf to be associated with false positivity of the ICT test. Due to suspicion that FTS positivity could be caused by a possible cross-reaction between *L. loa* and *W. bancrofti* infection, two methods (Microscopy and qPCR), confirmed the absence of *W. bancrofti* and demonstrated the presence of *L. loa*. As a consequence, there was need to assess the intensity of *L loa* in FTS positive individuals.

The overall *L. loa* Mf prevalence was 12.5% and the prevalence in males was higher than in females which are in agreement with previous works [13] but lower than recent reports [17]. This variation can be explained by the difference in study design and also the difference in geographical distribution of the disease. The prevalence in adults (> 15 years), was higher than that in children (< 15 years) which is in agreement with studies by Thompson et al. [24]. This can be explained by the type of activities people in the different age groups carry out that exposes them to the vectors of the parasite, like farming and hunting.

The FTS identified 1.6% (233/14446) persons as positive, implying that these individuals had CFA for *W. bancrofti,* however, further investigation of these FTS positive individuals by microscopy, using their night blood TBF, revealed that they had no mf of *W. bancrofti*, but those of *L. loa*, which is known to be endemic in the rain forest of Africa. This finding suggested a relationship between FTS positivity and *L. loa*, rather than LF infection in these districts. Results of the molecular analysis by qPCR confirmed the presence of *L. loa* and the absence of *W. bancofti.*

Logistic regression analyses indicated an association between FTS positivity and *L. Loa* mf loads. There was a linear relationship between FTS positivity and *L loa* mf load in agreement with previous reports and later demonstrated with the ICT [13, 17, 23]. FTS uses the same principal reagents as the ICT to detect *W. bancrofti* CFA and hence have the same specificity [16]. A possible explanation could be that the CFA detected by the FTS may be produced by *L. loa* and other pathogenic filariae parasites like *Onchocerca volvulus*, *M. perstans* [25], *B. malayi*. Also, the ICT components are similar with those of FTS and include monoclonal antibodies (AD12), originally produced by immunizing mice with antigens from the dog heartworm *Dirofilaria immitis*. The ability of this antigen to bind to other filaria species have been reported [7, 26]. Again, all levels of *L loa* are capable of inducing ICT positivity [13].

Comparative analyses between the different levels of endemicity with *L. loa* mf load, showed a higher risk of being detected FTS positive in individuals with very high loads of *L loa* mf. The Odds ratio in individuals with 8001–20,000 mf/ml of blood was 53.2 but as the mf load increased the odds ratio tripled in individuals with mf load greater than 30,000, to 151.9. This trend had earlier been reported [14, 27], which could serve as proof of association between FTS positivity and *L. loa* mf load.

## Conclusion

Our findings showed that the FTS test cross reacted with *L. loa* and its positivity was not associated to the presence of *W. bancrofti* in this area of Cameroon. A strong relationship was established between FTS positivity and *L. loa* TBF positivity. It could be concluded that *W. bancrofti* is absent from the 31 health districts situated in areas endemic for *L. loa* in the forest zones of Cameroon and LF MDA is therefore not necessary in these districts.

## Supplementary information

**Additional file 1: Figure S1.** Selection of the study sites. **Figure S2.** Map of the study area. **Figure S3.** Distribution of participants according to age and gender. **Figure S4.** Overall prevalence of lymphedema in the study area. **Figure S5.** Relationship between the proportion of positive FTS and the prevalence of *Loa loa* microfilaremia in 31 HDs of Cameroon. **Figure S6.** FTS positivity and *Loa loa* prevalence. **Table S1.** Infection profile for FTS, lymphedema and *loa loa* mf, in the 31 health districts. **Table S2.** Gender and age related prevalence of FTS, lymphedema and diurnal microfilaramia. **Table S3.** Distribution of FTS positivity across health districts, gender and age groups. **Table S4.** Logistic regression analysis of FTS results according to *L loa* load among MF carriers. **Table S5.** Comparison of day and night parasitological indices in lymphedema cases. **Table S6.** The relationship between FTS positivity and *Loa loa* infection intensity. **Figure S7.** Plot of log GMI (Mf/ml) of loasis night against day. **Figure S8.** Relationship between the proportion of positive FTS and the GMI of *L loa* mf densities (mf/ml) in 31 HDs of Cameroon. **Figure S9.** Prevalence of *Loa loa* microscopy at night and qPCR. **Table S7.** FTS positivity (%) in the 31 Health Districts. **Table S8.** Prevalence of *Loa loa* among age groups and across gender. **Table S9.** prevalence of *Loa loa* microfilaria loads (GMI mf/ml) for FTS positive individuals during the day and at Night. **Table S10.** Logistic regression analysis of FTS results according *L loa* load among MF carriers. **Table S11.** Comparing molecular (qPCR) with parasitological (Microscopy) of FTS positive individuals in the 31 health districts.

## Data Availability

All data used for this manuscript are either available in this published article and its supplementary information files.

## Abbreviations

CFA: Circulating filarial antigen
DBS: Dry blood spot
FTS: Filariasis test strip
GMI: Geometric mean intensity
HDs: Health districts
ICT: Immunochromatographic test
LF: Lymphatic filariasis
MDA: Mass drug administration
Mf: Microfilaria
PC: Preventive chemotherapy
qPCR: Real time polymerase chain reaction
SAE: Severe adverse events
TBF: Thick blood film

## References

[1] Bakajika DK, Nigo MM, Lotsima JP, Masikini GA, Fischer K, Lloyd MM, Weil GJ, Fischer PU (2014). Filarial antigenemia and Loa loa night blood microfilaremia in an area without bancroftian filariasis in the Democratic Republic of Congo. Am J Trop Med Hyg.

[2] Boussinesq M. La filariose lymphatique au Cameroun: état des connaissances**. ,** . In*.*, vol. 32. Bulletin de Liaison et de Documentation-OCEAC; 1999: 7–12.

[3] Boussinesq M, Kamgno J, S. P. Treatment of Loa patients: pre- and post- treatment loamicrofilarial levels and SAEs—experience in Cameroon. In., vol. 63rd annual meeting. Sheraton New Orleans Hotel, New Orleans, LA USA: ASTMH; 2014.

[4] WHO: Lymphatic filariasis. World Health Organization Fact Sheet 102**.** 2014.

[5] WHO: Lymphatic filariasis. World Health Organization Fact Sheet of 2nd March 2020. In*.*; 2020.

[6] Michael E, Malecela-Lazaro MN, Kabali C, Snow LC, Kazura JW (2006). Mathematical models and lymphatic filariasis control: endpoints and optimal interventions. Trends Parasitol.

[7] WHO: World Health Organization: Global programme to eliminate lymphatic filariasis: progress report, 2011. In*.*, vol. 87. Weekly Epidemiol Rec, ; 2012: 346–356. 22977953

[8] Nana-Djeunga HC, Tchatchueng-Mbougua JB, Bopda J, Mbickmen-Tchana S, Elong-Kana N, Nnomzo'o E, Akame J, Tarini A, Zhang Y, Njiokou F (2015). Mapping of Bancroftian Filariasis in Cameroon: prospects for elimination. PLoS Negl Trop Dis.

[9] Nana-Djeunga HC, Tchouakui M, Njitchouang GR, Tchatchueng-Mbougua JB, Nwane P, Domche A, Bopda J, Mbickmen-Tchana S, Akame J, Tarini A (2017). First evidence of lymphatic filariasis transmission interruption in Cameroon: Progress towards elimination. PLoS Negl Trop Dis.

[10] Esum M, Wanji S, Tendongfor N, Enyong P (2001). Co-endemicity of loiasis and onchocerciasis in the South West Province of Cameroon: implications for mass treatment with ivermectin. Trans R Soc Trop Med Hyg.

[11] Molyneux DH, Hopkins A, Bradley MH, Kelly-Hope LA (2014). Multidimensional complexities of filariasis control in an era of large-scale mass drug administration programmes: a can of worms. Parasit Vectors.

[12] Kelly-Hope LA, Hemingway J, Taylor MJ, Molyneux DH (2018). Increasing evidence of low lymphatic filariasis prevalence in high risk Loa loa areas in central and West Africa: a literature review. Parasit Vectors.

[13] Wanji S, Kengne-Ouafo JA, Esum ME, Chounna PW, Tendongfor N, Adzemye BF, Eyong JE, Jato I, Datchoua-Poutcheu FR, Kah E (2015). Situation analysis of parasitological and entomological indices of onchocerciasis transmission in three drainage basins of the rain forest of South West Cameroon after a decade of ivermectin treatment. Parasit Vectors.

[14] Wanji S, Amvongo-Adjia N, Njouendou AJ, Kengne-Ouafo JA, Ndongmo WP, Fombad FF, Koudou B, Enyong PA, Bockarie M (2016). Further evidence of the cross-reactivity of the Binax NOW(R) Filariasis ICT cards to non-Wuchereria bancrofti filariae: experimental studies with Loa loa and Onchocerca ochengi. Parasit Vectors.

[15] Pion SD, Montavon C, Chesnais CB, Kamgno J, Wanji S, Klion AD, Nutman TB, Boussinesq M (2016). Positivity of antigen tests used for diagnosis of lymphatic Filariasis in individuals without Wuchereria bancrofti infection but with high Loa loa Microfilaremia. Am J Trop Med Hyg.

[16] Weil GJ, Curtis KC, Fakoli L, Fischer K, Gankpala L, Lammie PJ, Majewski AC, Pelletreau S, Won KY, Bolay FK (2013). Laboratory and field evaluation of a new rapid test for detecting Wuchereria bancrofti antigen in human blood. Am J Trop Med Hyg.

[17] Wanji S, Esum ME, Njouendou AJ, Mbeng AA, Chounna Ndongmo PW, Abong RA, Fru J, Fombad FF, Nchanji GT, Ngongeh G (2019). Mapping of lymphatic filariasis in loiasis areas: a new strategy shows no evidence for Wuchereria bancrofti endemicity in Cameroon. PLoS Negl Trop Dis.

[18] Rao RU, Atkinson LJ, Ramzy RM, Helmy H, Farid HA, Bockarie MJ, Susapu M, Laney SJ, Williams SA, Weil GJ (2006). A real-time PCR-based assay for detection of Wuchereria bancrofti DNA in blood and mosquitoes. Am J Trop Med Hyg.

[19] Fink DL, Kamgno J, Nutman TB (2011). Rapid molecular assays for specific detection and quantitation of Loa loa microfilaremia. PLoS Negl Trop Dis.

[20] Natural Earth: Natural Earth. Available at https://www.naturalearthdata.com/. Accessed 02 Novemebr **,**2019**.**.

[21] Yahathugoda TC, Supali T, Rao RU, Djuardi Y, Stefani D, Pical F, Fischer PU, Lloyd MM, Premaratne PH, Weerasooriya MV (2015). A comparison of two tests for filarial antigenemia in areas in Sri Lanka and Indonesia with low-level persistence of lymphatic filariasis following mass drug administration. Parasit Vectors.

[22] More SJ, Copeman DB (1990). A highly specific and sensitive monoclonal antibody-based ELISA for the detection of circulating antigen in bancroftian filariasis. Trop Med Parasitol.

[23] Pion SD, Montavon C, Chesnais CJ, Kamgno J, Wanji. S ea. Correlation between high Loa loa microfilaremia and levels of circulating filarial antigens used to detect Wuchereriabancrofti infection. In: *63rd Annual Meeting, TropMed14.* Sheraton New Hotel NewOrleans LAUSA: ASTMH; 2014: pp. 377.

[24] Thomson MC, Obsomer V, Kamgno J, Gardon J, Wanji S, Takougang I, Enyong P, Remme JH, Molyneux DH, Boussinesq M (2004). Mapping the distribution of Loa loa in Cameroon in support of the African Programme for Onchocerciasis control. Filaria J.

[25] Deribe K, Beng AA, Cano J, Njouendo AJ, Fru-Cho J, Awah AR, Eyong ME, Chounna Ndongmo PW, Giorgi E, Pigott DM (2018). Mapping the geographical distribution of podoconiosis in Cameroon using parasitological, serological, and clinical evidence to exclude other causes of lymphedema. PLoS Negl Trop Dis.

[26] Weil GJ, Ramzy RM (2007). Diagnostic tools for filariasis elimination programs. Trends Parasitol.

[27] Ottesen EA, Duke BO, Karam M, Behbehani K (1997). Strategies and tools for the control/elimination of lymphatic filariasis. Bull World Health Organ.

