# Supplementary material for: Mapping lymphatic filariasis in Loa loa endemic health districts naïve for ivermectin mass administration and situated in the forested zone of Cameroon
Source: BMC Infect Dis. 2020 Apr 16;20:284. doi: 10.1186/s12879-020-05009-3 (PMC7164349; doi:10.1186/s12879-020-05009-3)
Supplement: Supplementary file 1 — Additional file 1: Figure S1. Selection of the study sites. Figure S2. Map of the study area. Figure S3. Distribution of participants according to age and gender. Figure S4. Overall prevalence of lymphedema in the study area. Figure S5. Relationship between the proportion of positive FTS and the prevalence of Loa loa microfilaremia in 31 HDs of Cameroon. Figure S6. FTS positivity and Loa loa prevalence. Table S1. Infection profile for FTS, lymphedema and loa loa mf, in the 31 health districts. Table S2. Gender and age related prevalence of FTS, lymphedema and diurnal microfilaramia. Table S3. Distribution of FTS positivity across health districts, gender and age groups. Table S4. Logistic regression analysis of FTS results according to L loa load among MF carriers. Table S5. Comparison of day and night parasitological indices in lymphedema cases. Table S6. The relationship between FTS positivity and Loa loa infection intensity. Figure S7. Plot of log GMI (Mf/ml) of loasis night against day. Figure S8. Relationship between the proportion of positive FTS and the GMI of L loa mf densities (mf/ml) in 31 HDs of Cameroon. Figure S9. Prevalence of Loa loa microscopy at night and qPCR. Table S7. FTS positivity (%) in the 31 Health Districts. Table S8. Prevalence of Loa loa among age groups and across gender. Table S9. prevalence of Loa loa microfilaria loads (GMI mf/ml) for FTS positive individuals during the day and at Night. Table S10. Logistic regression analysis of FTS results according L loa load among MF carriers. Table S11. Comparing molecular (qPCR) with parasitological (Microscopy) of FTS positive individuals in the 31 health districts. [file 12879_2020_5009_MOESM1_ESM.doc]

**Supplementary material**

Forest Zone of Cameroon

31 HDs, 124 Communities

East Region

Center Region

Littoral Region

South Region

13 HDs

10 HDs

04 HDs

04 HDs

52 villages

40 villages

16 villages

16 villages

**Figure S1: Selection of the study sites**


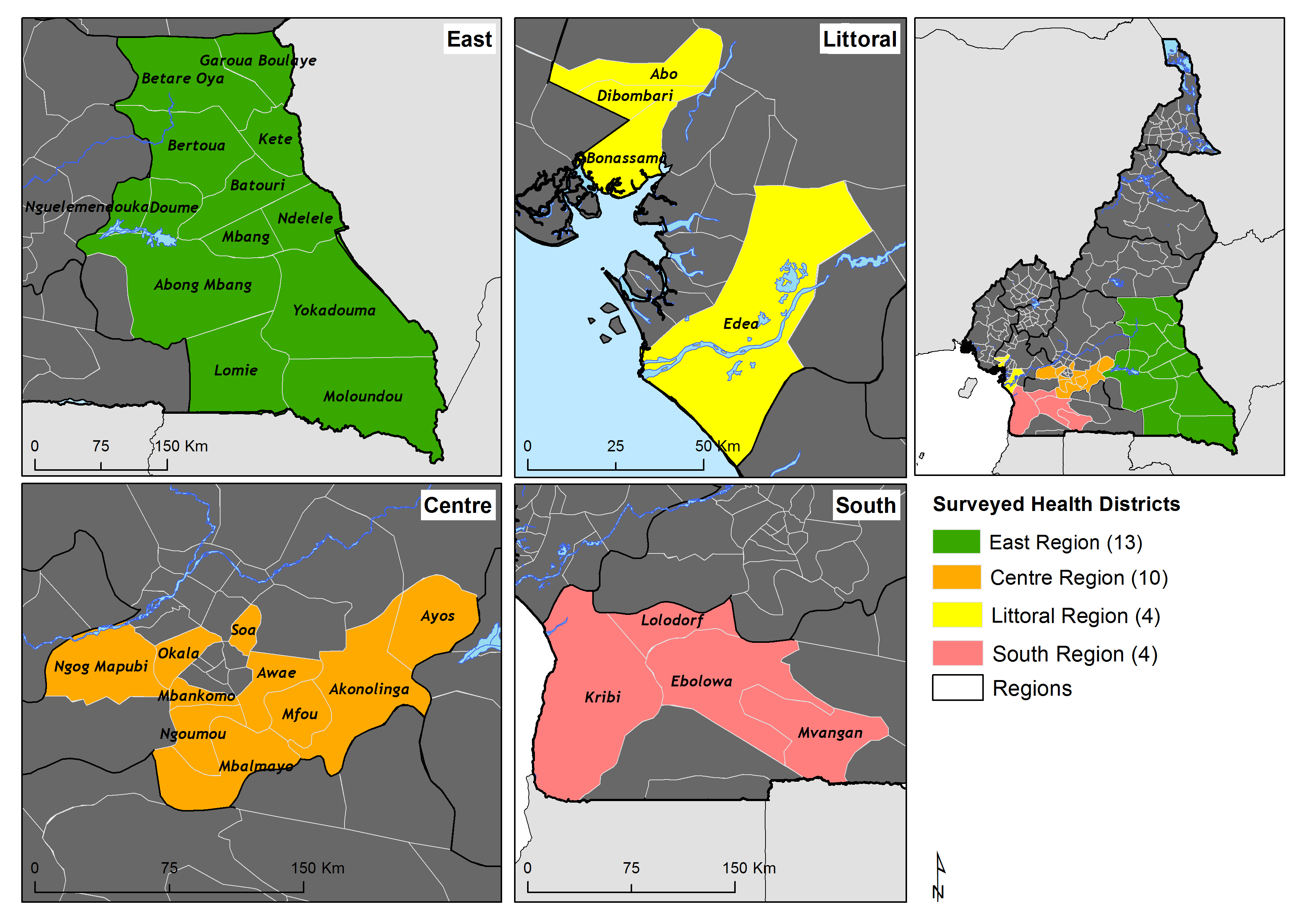


Figure S2: Map of the study area.


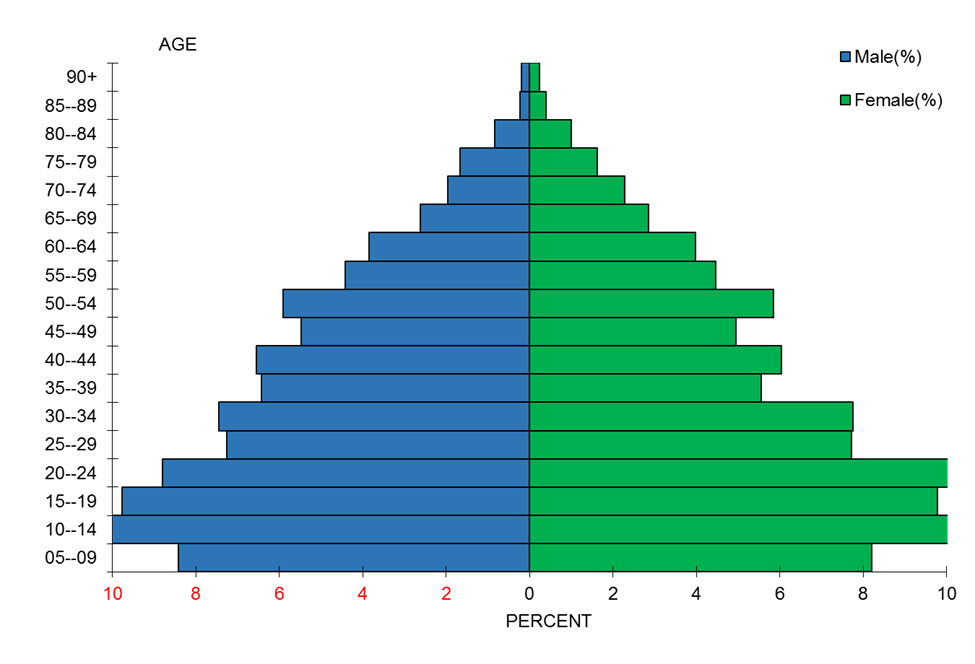


**Figure S3: Distribution of participants according to age and gender**


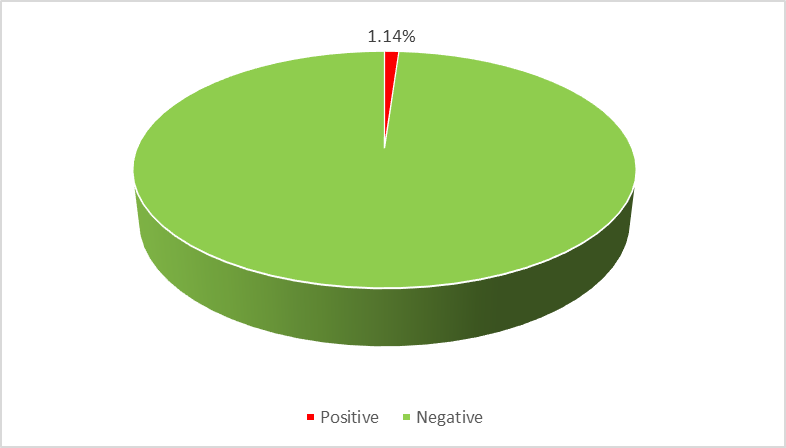


**Figure S4: Overall prevalence of lymphedema in the study area**

**
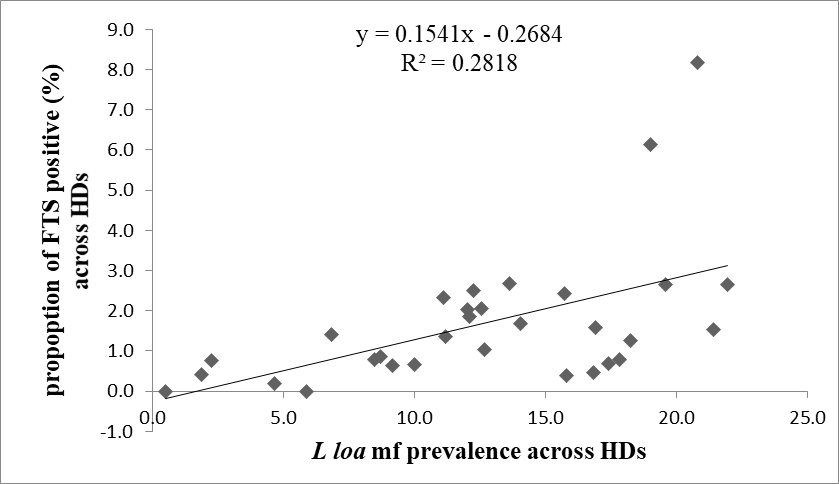
**

**Figure S5: Relationship between the proportion of positive FTS and the prevalence of *Loa loa* microfilaremia in 31 HDs of Cameroon.**

**
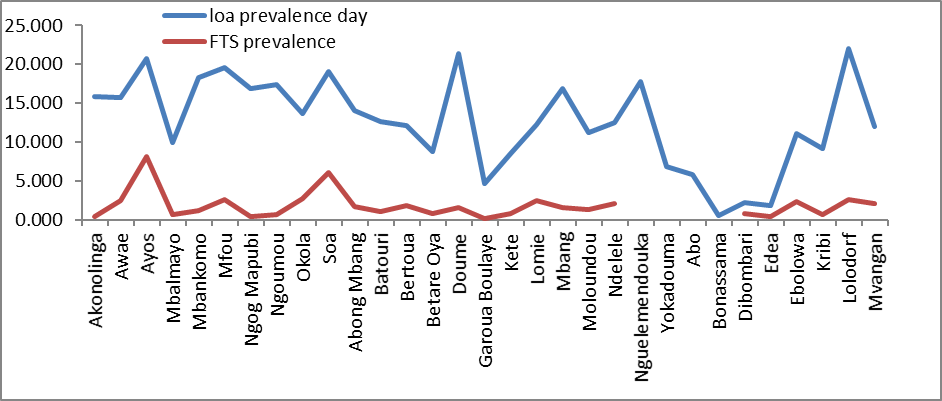
**

## Figure S6: FTS positivity and *Loa loa* prevalence.

| **Table S1. Infection profile for FTS, lymphedema and *loa loa* mf, in the 31 health districts.** | | | | | | | | | | |
| --- | --- | --- | --- | --- | --- | --- | --- | --- | --- | --- |
| **Parameter** | **District** | **Examined participants**  **(N)** | **FTS**  **+ve (N)** | **FTS prev.**  **(%)** | **Lymphedema +ves (N)** | **Lymphedema.-prev.(%)** | ***L. loa* +ves**  **N** | ***L. loa* prev.**  **(%)** | ***L. loa* GMI (mf/ml)** | |
|  |  |  |  |  |  |  |  |  | **Day** | **Night** |
| **Gender** | **Males** | **7221** | **141** | **2,0** | **82** | **1.1** | ***1071*** | ***14.8*** |  |  |
|  | **Females** | **7225** | **92** | **1.3** | **81** | **1.1** | ***734*** | ***10.2*** |  |  |
| **Age -group** | **Adults (≥15Yrs)** | **10295** | **218** | **2.1** | **147** | **1.4** | ***1624*** | ***15.8*** |  |  |
|  | **Children (5-14 Yrs)** | **4151** | **15** | **0.4** | **16** | **0.4** | ***181*** | ***4.8*** |  |  |
| EAST | Abong mbang | 534 | 9 | 1.7 | 6 | 1.1 | 75 | 14.0 | 1017.7 | 84.7 |
|  | Batouri | 387 | 4 | 1 | 11 | 2.8 | 49 | 12.7 | 1241.6 | 304 |
|  | Bertoua | 487 | 9 | 1.8 | 8 | 1.6 | 59 | 12.1 | 2761.6 | 154.3 |
|  | Betare oya | 470 | 4 | 0.9 | 1 | 0.2 | 41 | 8.7 | 115 | 59.6 |
|  | Doume | 523 | 8 | 1.5 | 5 | 1 | 112 | 21.4 | 11058.3 | 112.1 |
|  | Garoua boulaye | 534 | 1 | 0.2 | 2 | 0.4 | 25 | 4.7 | 0 | 0 |
|  | Kete | 508 | 4 | 0.8 | 5 | 1 | 43 | 8.5 | 514.2 | 3.6 |
|  | Lomie | 481 | 12 | 2.5 | 0 | 0 | 59 | 12.3 | 83.8 | 37.4 |
|  | Mbang | 503 | 8 | 1.6 | 36 | 7.2 | 85 | 16.9 | 2715.1 | 487.1 |
|  | Moloundou | 519 | 7 | 1.3 | 1 | 0.2 | 58 | 11.2 | 1240.7 | 111.4 |
|  | Ndelelle | 486 | 10 | 2.1 | 2 | 0.4 | 61 | 12.6 | 653 | 32.8 |
|  | Ngoulemendouka | 516 | 4 | 0.8 | 1 | 0.2 | 92 | 17.8 | 105.2 | 13.5 |
|  | Yokadouma | 496 | 7 | 1.4 | 5 | 1 | 34 | 6.9 | 103.6 | 4.5 |
| **Total** |  | **6444** | **87** | **1.4** | **83** | **1.3** | **793** | **12.3** | **621.3** | **61** |
|  |  |  |  |  |  |  |  |  |  |  |
| CENTER | Akonolinga | 519 | 2 | 0.4 | 0 | 0 | 82 | 15.8 | 6956.1 | 1553.3 |
|  | Awae | 451 | 11 | 2.4 | 5 | 1.1 | 71 | 15.7 | 312.7 | 47.7 |
|  | Ayos | 452 | 37 | 8.2 | 20 | 4.4 | 94 | 20.8 | 17.2 | 3 |
|  | Mbalmayo | 460 | 3 | 0.7 | 0 | 0 | 46 | 10 | 17675.4 | 296.7 |
|  | Mbankomo | 477 | 6 | 1.3 | 0 | 0 | 87 | 18.2 | 2894.9 | 226 |
|  | Mfou | 490 | 13 | 2.7 | 10 | 2 | 96 | 19.6 | 1932.9 | 108.4 |
|  | Ngogmapoubi | 422 | 2 | 0.5 | 1 | 0.2 | 71 | 16.8 | 0 | 0 |
|  | Ngoumou | 431 | 3 | 0.7 | 2 | 0.5 | 75 | 17.4 | 783.8 | 33.9 |
|  | Okola | 447 | 12 | 2.7 | 15 | 3.4 | 61 | 13.7 | 37.9 | 7.3 |
|  | Soa | 326 | 20 | 6.1 | 11 | 3.4 | 62 | 19.0 | 535.4 | 56.1 |
| **Total** |  | **4475** | **109** | **2.4** | **64** | **1.4** | **745** | **16.6** | **210** | **18.9** |
|  |  |  |  |  |  |  |  |  |  |  |
| SOUTH | Ebolowa | 513 | 12 | 2.3 | 0 | 0 | 57 | 11.1 | 205.2 | 44.4 |
|  | Kribi | 470 | 3 | 0.6 | 4 | 0.9 | 43 | 9.2 | 74 | 0 |
|  | Lolordolf | 264 | 7 | 2.7 | 0 | 0 | 58 | 22 | 2690. | 106.9 |
|  | Mvangan | 491 | 10 | 2 | 0 | 0 | 59 | 12.1 | 216. | 28.4 |
| **Total** |  | **1738** | **32** | **1.8** | **4** | **0.2** | **217** | **12.5** | **344.3** | **44.6** |
|  |  |  |  |  |  |  |  |  |  |  |
| LITTORAL | Abo | 510 | 0 | 0 | 6 | 1.2 | 30 | 5.9 | - |  |
|  | Bonassama | 404 | 0 | 0 | 3 | 0.7 | 2 | 0.5 |  | -- |
|  | Dibombari | 397 | 3 | 0.8 | 0 | 0 | 9 | 2.3 | 23.7 | 0 |
|  | Edea | 478 | 2 | 0.4 | 3 | 0.6 | 9 | 1.9 | 0 | 0 |
| **Total** |  | **1789** | **5** | **0.3** | **12** | **0.8** | **50** | **2.7** | **5.9** | **0** |
| **OVERALL** |  | **14446** | **233** | **1.6** | **163** | **1.14** | **1805** | **12.5** | **311** | **30.9** |

**Table S2.** Gender and age related prevalence of FTS, lymphedema and diurnal microfilaramia.

| **Variable** | **No examined** | **FTS positive (%)** | **Lymphedema positive** | ***Loa loa* positive /(%)** | ***Loa loa* GMI** | ***#P-value for FTS*** | ****P-value for L.loa*** |
| --- | --- | --- | --- | --- | --- | --- | --- |
| Gender |  |  |  |  |  |  |  |
| **Males** | 7221 | 141 (2.0) | 82(1.1) | 1071(14.8) | 2.9 | P < 0.05 | p<0.001 |
| **Female** | 7225 | 92 (1.3) | 81(1.1) | 734 (10.2) | 2.9 |  |
| Age group |  |  |  |  |  |  |  |
| **Adults (≥15)** | 10295 | 218 (2.1) | 147(1.4) | 1624 (15.8) |  | P < 0.05 | p<0.001 |
| **Children (5-15)** | 4151 | 15 (0.4) | 16(0.4) | 181 (4.8) |  |  |
| **5-20** | 3659 | 72(2.0) | 26(0.7) | 417(11.4) | 2.8 |  |  |
| **21-36** | 2681 | 69(2.6) | 40(1.5) | 461(17.2) | 2.9 |  |  |
| **37-52** | 5591 | 22(0.4) | 25(0.5) | 290(5.2) | 2.9 |  |  |
| **53-68** | 1903 | 44(2.3) | 49(2.6) | 450(23.6) | 3.4 |  |  |
| **69-84** | 722 | 28(3.9) | 21(2.9) | 182(25.2) | 1.8 |  |  |
| **85+** | 79 | 1(1.3) | 2(2.5) | 11(!3.9) | 2.3 |  |  |

### Table S3: Distribution of FTS positivity across health districts, gender and age groups

| **Region** | **District** | **overall** | | **Age group** | | | | **Sex** | | | |
| --- | --- | --- | --- | --- | --- | --- | --- | --- | --- | --- | --- |
|  |  |  |  | **Adults (>15 years)** | | **Children (5-15 years)** | | **Females** | | **Males** | |
|  |  | **Exa-mined** | **FTS(%) positive** | **Exami-ned** | **FTS positive** | **Exami-ned** | **FTS positive** | **Exami-ned** | **FTS positive** | **Exami-ned** | **FTS positive** |
| **Center** | Akonolinga | 519 | 0.4 | 343 | 2 | 176 | 0 | 269 | 1 | 250 | 1 |
|  | Awae | 451 | **2.4** | 356 | 11 | 95 | 0 | 227 | 6 | 224 | 5 |
|  | Ayos | 452 | **8.2** | 331 | 32 | 121 | 5 | 238 | 16 | 214 | 21 |
|  | Mbalmayo | 460 | 0.7 | 299 | 2 | 161 | 1 | 231 | 0 | 229 | 3 |
|  | Mbankomo | 477 | **1.3** | 330 | 6 | 147 | 0 | 236 | 4 | 241 | 2 |
|  | Mfou | 490 | **2.7** | 400 | 13 | 90 | 0 | 257 | 5 | 233 | 8 |
|  | Ngog Mapubi | 422 | 0.5 | 325 | 2 | 97 | 0 | 209 | 1 | 213 | 1 |
|  | Ngoumou | 431 | 0.7 | 330 | 2 | 101 | 1 | 234 | 1 | 197 | 2 |
|  | Okola | 447 | **2.7** | 362 | 12 | 85 | 0 | 215 | 5 | 232 | 7 |
|  | Soa | 326 | **6.1** | 236 | 19 | 90 | 1 | 201 | 8 | 125 | 12 |
|  | **Total** | **4475** |  | **3312** | **101** | **1163** | **8** | **2317** | **47** | **2158** | **62** |
|  | **Prevalence** | **-** | **2.4%** | **-** | **3.0%** | **-** | 0.7% | **-** | **2.0%** | **-** | **2.9%** |
|  | p. value |  |  |  | <0.001 |  |  |  | <0.066 |  |  |
| **East** | Abong Mbang | 534 | **1.7** | 344 | 7 | 190 | 2 | 259 | 2 | 275 | 7 |
|  | Batouri | 387 | **1.0** | 288 | 4 | 99 | 0 | 208 | 1 | 179 | 3 |
|  | Bertoua | 487 | **1.8** | 331 | 8 | 156 | 1 | 229 | 4 | 258 | 5 |
|  | Betare Oya | 470 | 0.9 | 292 | 2 | 178 | 2 | 214 | 2 | 256 | 2 |
|  | Doume | 523 | **1.5** | 334 | 8 | 189 | 0 | 242 | 4 | 281 | 4 |
|  | Garoua Boulaye | 534 | 0.2 | 321 | 1 | 213 | 0 | 273 | 1 | 261 | 0 |
|  | Kete | 508 | 0.8 | 361 | 4 | 147 | 0 | 199 | 0 | 309 | 4 |
|  | Lomie | 481 | **2.5** | 338 | 12 | 143 | 0 | 258 | 6 | 223 | 6 |
|  | Mbang | 503 | **1.6** | 381 | 8 | 122 | 0 | 242 | 3 | 261 | 5 |
|  | Moloundou | 519 | **1.3** | 416 | 7 | 103 | 0 | 212 | 3 | 307 | 4 |
|  | Ndelele | 486 | **2.1** | 300 | 9 | 186 | 1 | 209 | 4 | 277 | 6 |
|  | Nguelemendouka | 516 | 0.8 | 343 | 4 | 173 | 0 | 300 | 3 | 216 | 1 |
|  | Yokadouma | 496 | **1.4** | 390 | 7 | 106 | 0 | 302 | 1 | 194 | 6 |
|  | **Total** | **6444** |  | **4439** | **81** | **2005** | **6** | **3147** | **34** | **3297** | **53** |
|  | **Prevalence** | **-** | **1.40%** | **-** | **1.80%** | **-** | **0.30%** | **-** | **1.10%** | **-** | **1.60%** |
|  | p. value |  |  |  | <0.001 |  |  |  |  | <0.067 |  |
| **Littoral** | Abo | 510 | 0.0 | 421 | 0 | 89 | 0 | 255 | 0 | 255 | 0 |
|  | Bonassama | 404 | 0.0 | 289 | 0 | 115 | 0 | 227 | 0 | 177 | 0 |
|  | Dibombari | 397 | 0.8 | 246 | 3 | 151 | 0 | 214 | 1 | 183 | 2 |
|  | Edea | 478 | 0.4 | 305 | 2 | 173 | 0 | 240 | 1 | 238 | 1 |
|  | **Total** | **1789** |  | **1261** | **5** | **528** | **0** | **936** | **2** | **853** | **3** |
|  | **Prevalence** | **-** | **0.30%** | **-** | **0.40%** | **-** | **0.00%** | **-** | **0.20%** | **-** | **0.40%** |
|  | p. value |  |  |  | <0.330 |  |  |  | <0.067 |  |  |
| **South** | Ebolowa | 513 | **2.3** | 335 | 11 | 178 | 1 | 250 | 4 | 263 | 8 |
|  | Kribi | 470 | 0.6 | 357 | 3 | 113 | 0 | 222 | 1 | 248 | 2 |
|  | Lolodorf | 264 | **2.7** | 216 | 7 | 48 | 0 | 113 | 1 | 151 | 6 |
|  | Mvangan | 491 | **2.0** | 375 | 10 | 116 | 0 | 240 | 3 | 251 | 7 |
|  | **Total** | **1738** |  | **1283** | **31** | **455** | **1** | **825** | **9** | **913** | **23** |
|  | **Prevalence** | **-** | **1.80%** | **-** | **2.40%** | **-** | **0.20%** | **-** | **1.10%** | **-** | **2.50%** |
|  | p. value |  |  |  | <0.001 |  |  |  | 0.674 |  |  |

**FTS positivity (%) in Bold (**
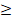
**1%).**

**Table S4: Logistic regression analysis of FTS results according to *L loa* load among MF carriers**

| **Region** | **Loa Mf load** | **Odds ratio** | **95% C.I OR** | **P. value** |
| --- | --- | --- | --- | --- |

|  | 0 | **Reference** | - | - |
| --- | --- | --- | --- | --- |
|  | 1-8000 | **6.1** | 4.4-8.6 | <0.001 |
| **Over all** | 8001-20000 | **53.2** | 35.2-80.4 | <0.001 |
| **All regions** | 20001-30000 | **103.1** | 55.4-192 | <0.001 |
|  | >30000 | **151.9** | 85.4-270.2 | <0.001 |
| **Sex** | female | **reference** | - | - |
|  | -male | **1.3** | 0.973-1.735 | **<0.075** |
| **Age group** | Children | - | - | - |
|  | -Adults | **3.395** | 1.942-5.935 | **<0.002** |

**Table S5.** Comparison of day and night parasitological indices in lymphedema cases

| Region | Number of lymphedema examined | **Day** | | **Night** | |
| --- | --- | --- | --- | --- | --- |
|  |  | Loa mf positive | Loa GMI (mf/ml) | Loa mf positive | Loa GMI (mf/ml) |
| Centre | 64 | 18(28.1) | 2.2 | 3(4.7) | 2.4 |
| East | 83 | 13(15.7) | 2.4 | 1(1.2) | 3.1 |
| Littoral | 15 | 2(16.7) | 3.2 | 0(0.0) | 0 |
| South | 4 | 1(25.0) | 2.1 | 0(0.0) | 0 |
| **Total** | **163** | **34(20.0)** | **9.9** | **4(2.4)** | **5.4** |

**Table S6. The relationship between FTS positivity and *Loa loa* infection intensity**.

| ***Loa loa* mf load**  **Mf/ml** | **Participants examined**  **N** | **FTS positives**  **N** | **Percentage %** |
| --- | --- | --- | --- |
| 0 | 12652 | 85 | 0.7 |
| 1 to 8,000 | 1530 | 59 | 3.9 |
| 8001-20,000 | 163 | 42 | 25.8 |
| 20001-30,000 | 47 | 19 | 40.4 |
| >30,000. | 54 | 27 | 50.0 |
| **TOTAL** | **14446** | **232*** | **1.6** |
| **Linear to Linear association Test** |  | **P <** | **0.002** |

*one positive FTS slide was lost


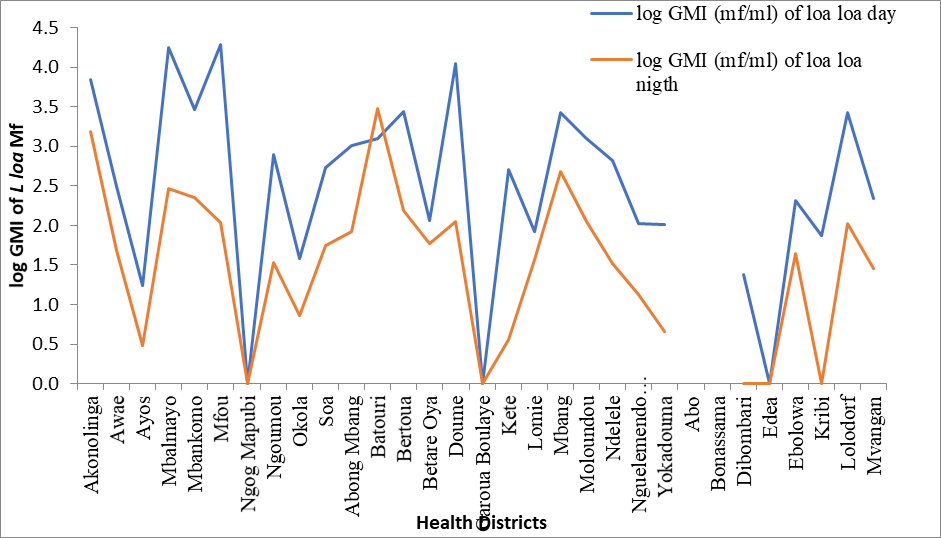


**Figure S7: Plot of log GMI (Mf/ml) of loasis night against day**


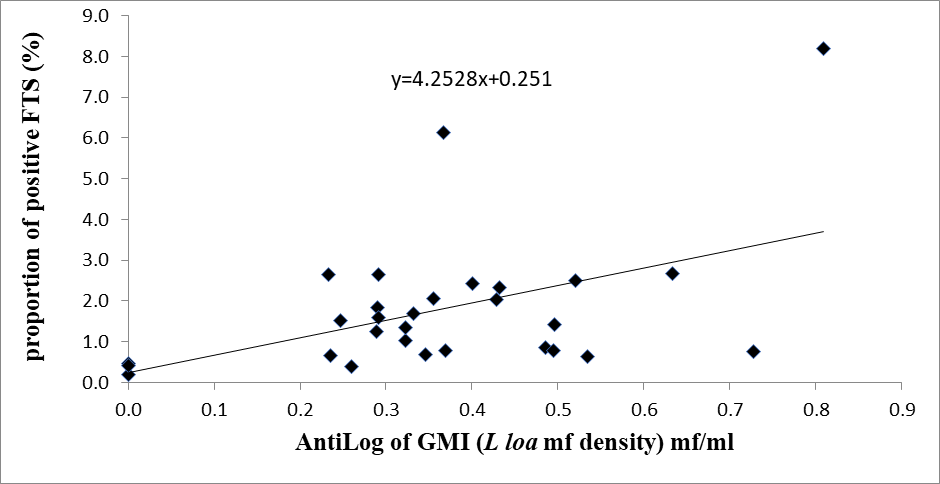


**Figure S8: Relationship between the proportion of positive FTS and the GMI of *L loa* mf densities (mf/ml) in 31 HDs of Cameroon**

**
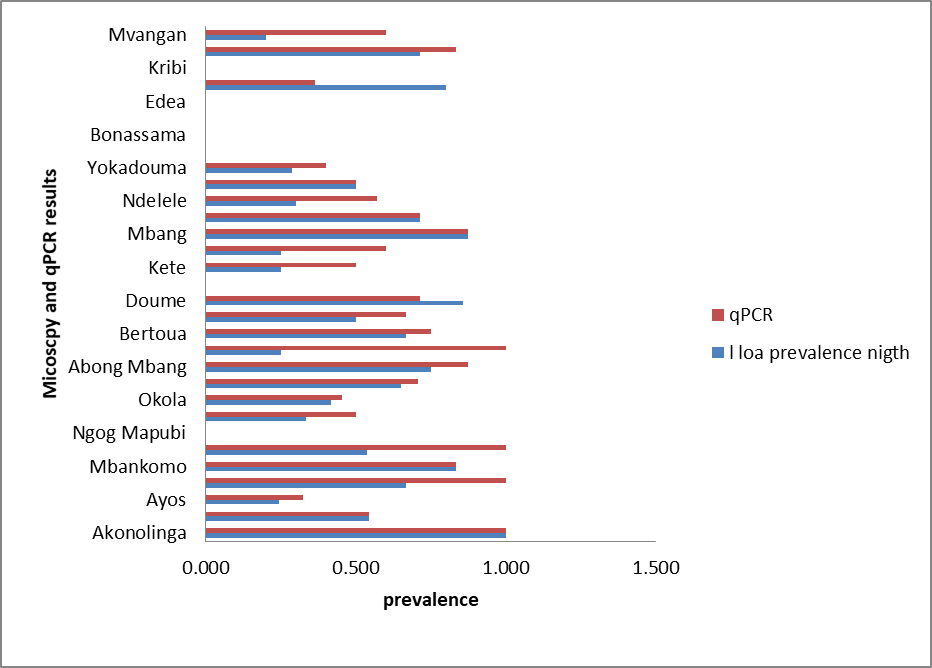
**

**Figure S9: Prevalence of *Loa loa* microscopy at night and qPCR**

| **Table S7: FTS positivity (%) in the 31 Health Districts** | | | | | |  | | |  |  |
| --- | --- | --- | --- | --- | --- | --- | --- | --- | --- | --- |
| **REGION** | **District** | **Examined Participants (N)** | | | | **FTS Positive (N)** | | **Prevalence (%)** |  | |
|  | Abong Mbang | | | 534 |  | 9 |  | 1.7 |  | |
|  | Batouri | |  | 387 |  | 4 |  | 1 |  | |
|  | Bertoua | |  | 487 |  | 9 |  | 1.8 |  | |
| **EAST** | Betare Oya | | | 470 |  | 4 |  | 0.9 |  | |
|  | Doume | |  | 523 |  | 8 |  | 1.5 |  | |
|  | Garoua Boulaye | | | 534 |  | 1 |  | 0.2 |  | |
|  | Kete | |  | 508 |  | 4 |  | 0.8 |  | |
|  | Lomie | |  | 481 |  | 12 |  | 2.5 |  | |
|  | Mbang | |  | 503 |  | 8 |  | 1.6 |  | |
|  | Moloundou | | | 519 |  | 7 |  | 1.3 |  | |
|  | Ndelele | |  | 486 |  | 10 |  | 2.1 |  | |
|  | Nguelemendouka | | | 516 |  | 4 |  | 0.8 |  | |
|  | Yokadouma | | | 496 |  | 7 |  | 1.4 |  | |
| **Overall(EAST region)** | | | | **6444** |  | **87** |  | **1.4** |  | |
|  |  | |  |  |  |  |  |  |  | |
|  | Akonolinga | | | 519 |  | 2 |  | 0.4 |  | |
|  | Awae | |  | 451 |  | 11 |  | 2.4 |  | |
|  | Ayos | |  | 452 |  | 37 |  | 8.2 |  | |
| **CENTER** | Mbalmayo | | | 460 |  | 3 |  | 0.7 |  | |
|  | Mbankomo | | | 477 |  | 6 |  | 1.3 |  | |
|  | Mfou | |  | 490 |  | 13 |  | 2.7 |  | |
|  | Ngog Mapubi | | | 422 |  | 2 |  | 0.5 |  | |
|  | Ngoumou | | | 431 |  | 3 |  | 0.7 |  | |
|  | Okola | |  | 447 |  | 12 |  | 2.7 |  | |
|  | Soa | |  | 326 |  | 20 |  | 6.1 |  | |
| **Overall(Center region)** | | | | **4475** |  | **109** |  | **2.4** |  | |
|  |  | |  |  |  |  |  |  |  | |
|  | Ebolowa | |  | 513 |  | 12 |  | 2.3 |  | |
| **SOUTH** | Kribi | |  | 470 |  | 3 |  | 0.6 |  | |
|  | Lolodorf | |  | 264 |  | 7 |  | 2.7 |  | |
|  | Mvangan | |  | 491 |  | 10 |  | 2 |  | |
| **Over all (South region)** | | | | **1738** |  | **32** |  | **1.8** |  | |
|  |  | |  |  |  |  |  |  |  | |
|  | Abo | |  | 510 |  | 0 |  | 0 |  | |
| **LITTORAL** | Bonassama | | | 404 |  | 0 |  | 0 |  | |
|  | Dibombari | | | 397 |  | 3 |  | 0.8 |  | |
|  | Edea | |  | 478 |  | 2 |  | 0.4 |  | |
| **Overall (Littoral region)** | | | | **1789** |  | **5** |  | **0.3** |  | |
| **Grand total** | | |  | **14446** |  | **233** |  | **1.61** |  | |

### Table S8: Prevalence of *Loa loa* among age groups and across gender

| **Region** | **District** | **Overall** | | **Females** | | **Males** | | **Adult**  **(>15 years)** | | **Children**  **(5-15 years)** | |
| --- | --- | --- | --- | --- | --- | --- | --- | --- | --- | --- | --- |
|  |  | **Exami-ned** | **Loa Positive** | **Exami-ned** | **Loa Positive** | **Exami-ned** | **Loa Positive** | **Exami-ned** | **Loa Positive** | **Exami-ned** | **Loa Positive** |
| **Center** | Akonolinga | 519 | 82 | 269 | 31 | 250 | 51 | 343 | 68 | 176 | 14 |
|  | Awae | 451 | 71 | 227 | 31 | 224 | 40 | 356 | 66 | 95 | 5 |
|  | Ayos | 452 | 94 | 238 | 46 | 214 | 48 | 331 | 86 | 121 | 8 |
|  | Mbalmayo | 460 | 46 | 231 | 23 | 229 | 23 | 299 | 42 | 161 | 4 |
|  | Mbankomo | 477 | 87 | 236 | 36 | 241 | 51 | 330 | 75 | 147 | 12 |
|  | Mfou | 490 | 96 | 257 | 39 | 233 | 57 | 400 | 90 | 90 | 6 |
|  | Ngog Mapubi | 422 | 71 | 209 | 31 | 213 | 40 | 325 | 64 | 97 | 7 |
|  | Ngoumou | 431 | 75 | 234 | 32 | 197 | 43 | 330 | 71 | 101 | 4 |
|  | Okola | 447 | 61 | 215 | 26 | 232 | 35 | 362 | 58 | 85 | 3 |
|  | Soa | 326 | 62 | 201 | 25 | 125 | 37 | 236 | 55 | 90 | 7 |
|  | **Total** | **4475** | 745 | **2317** | **320** | **2158** | **425** | **3312** | **675** | **1163** | **70** |
|  | **Prevalence** | - | **16.6%** | **-** | **13.8%** | **-** | **19.7%** | **-** | **20.4%** | **-** | **6.0%** |
|  | **p. value** |  |  |  |  | <0.001 |  |  | <0.001 |  |  |
| **East** | Abong Mbang | 533 | 75 | 259 | 38 | 275 | 37 | 344 | 63 | 190 | 12 |
|  | Batouri | 387 | 49 | 208 | 20 | 179 | 29 | 288 | 42 | 99 | 7 |
|  | Bertoua | 487 | 59 | 229 | 21 | 258 | 38 | 331 | 56 | 156 | 3 |
|  | Betare Oya | 470 | 41 | 214 | 18 | 256 | 23 | 292 | 37 | 178 | 4 |
|  | Doume | 522 | 112 | 242 | 49 | 281 | 63 | 334 | 86 | 189 | 26 |
|  | Garoua Boulaye | 534 | 25 | 273 | 17 | 261 | 8 | 321 | 18 | 213 | 7 |
|  | Kete | 508 | 43 | 199 | 10 | 309 | 33 | 361 | 42 | 147 | 1 |
|  | Lomie | 481 | 59 | 258 | 16 | 223 | 43 | 338 | 56 | 143 | 3 |
|  | Mbang | 503 | 85 | 242 | 36 | 261 | 49 | 381 | 81 | 122 | 4 |
|  | Moloundou | 519 | 58 | 212 | 15 | 307 | 43 | 416 | 56 | 103 | 2 |
|  | Ndelele | 486 | 61 | 209 | 24 | 277 | 37 | 300 | 54 | 186 | 7 |
|  | Nguelemen-douka | 516 | 92 | 300 | 51 | 216 | 41 | 343 | 76 | 173 | 16 |
|  | Yokadouma | 496 | 34 | 302 | 17 | 194 | 17 | 390 | 34 | 106 | 0 |
|  | **Total** | **6442** | 793 | **3147** | **332** | **3297** | **461** | **4439** | **701** | **2005** | **92** |
|  | **Prevalence** | - | **12.3%** | **-** | **10.5%** | **-** | **14.0%** | **-** | **15.8%** | **-** | **4.6%** |
|  | **p. value** |  |  |  |  | <0.001 |  |  | <0.001 |  |  |
| **Littoral** | Abo | 510 | 30 | 255 | 4 | 255 | 26 | 421 | 28 | 89 | 2 |
|  | Bonassama | 404 | 2 | 227 | 0 | 177 | 2 | 289 | 2 | 115 | 0 |
|  | Dibombari | 397 | 9 | 214 | 2 | 183 | 7 | 246 | 9 | 151 | 0 |
|  | Edea | 478 | 9 | 240 | 4 | 238 | 5 | 305 | 9 | 173 | 0 |
|  | **Total** | **1789** | 50 | **936** | **10** | **853** | **40** | **1261** | **48** | **528** | **2** |
|  | **Prevalence** | - | **2.8%** | **-** | **1.1%** | **-** | **4.7%** | **-** | **3.8%** | **-** | **0.4%** |
|  | **p.value** |  |  |  |  | <0.001 |  |  | <0.001 |  |  |
| **South** | Ebolowa | 511 | 57 | 250 | 23 | 263 | 34 | 335 | 51 | 178 | 6 |
|  | Kribi | 470 | 43 | 222 | 12 | 248 | 31 | 357 | 40 | 113 | 3 |
|  | Lolodorf | 264 | 58 | 113 | 17 | 151 | 41 | 216 | 56 | 48 | 2 |
|  | Mvangan | 491 | 59 | 240 | 19 | 251 | 40 | 375 | 53 | 116 | 6 |
|  | **Total** | **1736** | 217 | **825** | **71** | **913** | **146** | **1283** | **200** | **455** | **17** |
|  | **Prevalence** | - | **12.5%** | **-** | **8.6%** | **-** | **16.0%** | **-** | **15.6%** | **-** | **3.7%** |
|  | **p. value** |  |  |  |  | <0.001 |  |  | <0.001 |  |  |
| **Grand Total** | **N** | **14442** | 1805 | **7225** | **733** | **7221** | **1072** | **10295** | **1624** | **4151** | **181** |
|  | **Prevalence** | **-** | **12.5%** | **-** | **10.1%** | **-** | **14.8%** | **-** | **15.8%** | **-** | **4.4%** |
|  | **p. value** |  |  |  |  | <0.001 |  |  | <0.001 |  |  |

**Table S9: prevalence of *Loa loa*** microfilaria loads (GMI mf/ml) for FTS positive individuals during the day and at Night

|  |  |  | **Day** |  |  | **Night** |  |
| --- | --- | --- | --- | --- | --- | --- | --- |
| **Region** | District | Examined | *Loa loa* positive | *loa loa* Mf GMI (mf/ml) | Examined | *Loa loa* positive | *Loa loa* Mf GMI |
| **Center** | Akonolinga | 2 | 2 | 6956.1 | 2 | 2 | 1553.3 |
|  | Awae | 11 | 7 | 312.7 | 11 | 6 | 47.7 |
|  | Ayos | 37 | 13 | 17.2 | 36 | 9 | 3 |
|  | Mbalmayo | 3 | 3 | 17675.4 | 2 | 2 | 296.7 |
|  | Mbankomo | 6 | 5 | 2894.9 | 6 | 5 | 226 |
|  | Mfou | 13 | 13 | 19132.9 | 8 | 7 | 108.4 |
|  | Ngog Mapubi | 2 | 0 | 0 | 1 | 0 | 0 |
|  | Ngoumou | 3 | 3 | 783.8 | 2 | 1 | 33.9 |
|  | Okola | 12 | 5 | 37.9 | 12 | 5 | 7.3 |
|  | Soa | 20 | 14 | 535.4 | 17 | 13 | 56.1 |
|  | **Total** | **109** | **65** | **210** | **97** | **50** | **18.9** |
|  | **Percentage** | **-** | **59.60%** |  | **-** | **51.50%** |  |
| **East** | Abong Mbang | 8 | 7 | 1017.7 | 8 | 6 | 84.7 |
|  | Batouri | 4 | 3 | 1241.6 | 1 | 1 | 3040 |
|  | Bertoua | 9 | 7 | 2761.6 | 8 | 6 | 154.3 |
|  | Betare Oya | 4 | 2 | 115 | 3 | 2 | 59.6 |
|  | Doume | 7 | 7 | 11058.3 | 7 | 6 | 112.1 |
|  | Garoua Boulaye | 1 | 0 | 0 | 1 | 0 | 0 |
|  | Kete | 4 | 3 | 514.2 | 2 | 1 | 3.6 |
|  | Lomie | 12 | 6 | 83.8 | 6 | 3 | 37.4 |
|  | Mbang | 8 | 7 | 2715.1 | 8 | 7 | 487.1 |
|  | Moloundou | 7 | 5 | 1240.7 | 7 | 5 | 111.4 |
|  | Ndelele | 10 | 8 | 653 | 6 | 3 | 32.8 |
|  | Nguelemendouka | 4 | 2 | 105.2 | 4 | 2 | 13.5 |
|  | Yokadouma | 7 | 4 | 103.6 | 7 | 2 | 4.5 |
|  | Total | 85 | 61 | **621.3** | 68 | 44 | **61** |
|  | Percentage | **-** | **71.80%** | 0 | **-** | **64.70%** |  |
| **Littoral** | Dibombari | 3 | 1 | 23.7 | 1 | 0 | 0 |
|  | Edea | 2 | 0 | 0 | 1 | 0 | 0 |
|  | Total | 5 | 1 | **5.9** | 2 | 0 | **0** |
|  | Percentage | **-** | **20.00%** | 2690.3 | **-** | **0.00%** |  |
| **South** | Ebolowa | 10 | 6 | 205.2 | 11 | 8 | 44.4 |
|  | Kribi | 3 | 2 | 74 | 1 | 0 | 0 |
|  | Lolodorf | 7 | 6 | 2690.3 | 6 | 5 | 106.9 |
|  | Mvangan | 10 | 6 | 216.2 | 3 | 2 | 28.4 |
|  | Total | 30 | 20 | **344.3** | 21 | 15 | **44.6** |
|  | Percentage |  | 66.70% |  |  | 71.40% |  |
| **grand total** | **Percentage** |  | **64.20%** |  |  | **58.00%** |  |
|  |  | **229** | **147** | **311** | **188** | **109** | **30.9** |

### Table S10: Logistic regression analysis of FTS results according *L loa* load among MF carriers

| **Region** | **Loa Mf load** | **Odds ratio** | **95%C.I OR** | **P.value** |
| --- | --- | --- | --- | --- |
|  | 0 | **Reference** | - | - |
|  | 1-8,000 | **3.3** | 2.0-5.5 | <0.001 |
| **Center** | 8001-20000 | **32.9** | 18.3-59.2 | <0.001 |
|  | 20001-30000 | **45.3** | 16.1-128.1 | <0.001 |
|  | >30000 | **166.3** | 64.0-432.1 | <0.001 |
|  | 0 | **Reference** | - | - |
|  | 1-8000 | **8.8** | 5.0-15.7 | <0.001 |
| **East** | 8001-20000 | **73.9** | 36.5-149.9 | <0.001 |
|  | 20001-30000 | **178.3** | 71.3-446 | <0.001 |
|  | >30000 | **196.1** | 80.0-481.3 | <0.001 |
|  | 0 | **Reference** | - | - |
|  | 1-8000 | **8.9** | 3.7-21.8 | <0.001 |
| **South** | 8001-20000 | **41.7** | 13.0-134.5 | <0.001 |
|  | 20001-30000 | **225.4** | 33.9-1498.6 | <0.001 |
|  | >30000 | **42.9** | 7.9-232.7 | <0.001 |
|  | 0 | **Reference** | - | - |
|  | 1-8000 | **6.1** | 4.4-8.6 | <0.001 |
| **Over all** | 8001-20000 | **53.2** | 35.2-80.4 | <0.001 |
| **All regions** | 20001-30000 | **103.1** | 55.4-192 | <0.001 |
|  | >30000 | **151.9** | 85.4-270.2 | <0.001 |
| **Sex** | female | **reference** | - | - |
|  | -male | **1.3** | 0.973-1.735 | **<0.075** |
| **Age group** | Children | - | - | - |
|  | -Adults | **3.395** | 1.942-5.935 | **<0.002** |

### Table S11: Comparing molecular (qPCR) with parasitological (Microscopy) of FTS positive individuals in the 31 health districts.

| **Region** | **District** | **Examined** | **qPCR** | | **Microscopy** | |
| --- | --- | --- | --- | --- | --- | --- |
|  |  |  | ***W bancrofti*** | ***Loa loa*** | ***W bancrofti*** | ***Loa loa*** |
| **Center** | Akonolinga | 2 | 0 | 2 | 0 | 2 |
|  | Awae | 11 | 0 | 6 | 0 | 6 |
|  | Ayos | 37 | 0 | 12 | 0 | 9 |
|  | Mbalmayo | 2 | 0 | 2 | 0 | 2 |
|  | Mbankomo | 6 | 0 | 5 | 0 | 5 |
|  | Mfou | 3 | 0 | 3 | 0 | 3 |
|  | Ngog Mapubi | 1 | 0 | 0 | 0 | 0 |
|  | Ngoumou | 2 | 0 | 1 | 0 | 1 |
|  | Okola | 11 | 0 | 5 | 0 | 5 |
|  | Soa | 17 | 0 | 12 | 0 | 13 |
|  | **Total** | **92** | **0** | **48** | 0 | **46** |
|  | **Prevalence** | **-** | **0.0%** | **52.2%** | 0 | **50.0%** |
| **East** | Abong Mbang | 8 | 0 | 7 | 0 | 6 |
|  | Batouri | 1 | 0 | 1 | 0 | 1 |
|  | Bertoua | 8 | 0 | 6 | 0 | 6 |
|  | Betare Oya | 3 | 0 | 2 | 0 | 2 |
|  | Doume | 7 | 0 | 5 | 0 | 6 |
|  | Garoua Boulaye | 1 | 0 | 0 | 0 | 0 |
|  | Kete | 2 | 0 | 1 | 0 | 1 |
|  | Lomie | 5 | 0 | 3 | 0 | 3 |
|  | Mbang | 8 | 0 | 7 | 0 | 7 |
|  | Moloundou | 7 | 0 | 5 | 0 | 5 |
|  | Ndelele | 7 | 0 | 4 | 0 | 3 |
|  | Nguelemendouka | 2 | 0 | 1 | 0 | 1 |
|  | Yokadouma | 5 | 0 | 2 | 0 | 2 |
|  | **Total** | **64** | **0** | **44** | 0 | **43** |
|  | **Prevalence** | **-** | **0.0%** | **68.8%** | 0 | **67.2%** |
| **Littoral** | Dibombari | 2 | 0 | 0 | 0 | 0 |
|  | Edea | 1 | 0 | 0 | 0 | 0 |
|  | **Total** | **3** | **0** | **0** | 0 | **0** |
|  | **Prevalence** | **-** | **0.0%** | **0.0%** | 0 | **0.0%** |
| **South** | Ebolowa | 11 | 0 | 4 | 0 | 7 |
|  | Kribi | 1 | 0 | 0 | 0 | 0 |
|  | Lolodorf | 6 | 0 | 5 | 0 | 5 |
|  | Mvangan | 5 | 0 | 3 | 0 | 2 |
|  | **Total** | 23 | 0 | 12 | 0 | **14** |
|  | **Prevalence** | **-** | **0.0%** | **52.2%** | 0 | **60.9%** |
|  | **p. value** |  |  |  |  |  |
| **Grand Total** | **N** | **182** | **0** | **104** | 0 | **103** |
|  | **Prevalence** | **-** | **0.0%** | **57.1%** | 0 | **56.6%** |
|  | **p. value** | **0.999** |  |  |  |  |
